# CRISPR-Cas systems are present predominantly on mobile genetic elements in *Vibrio* species

**DOI:** 10.1186/s12864-019-5439-1

**Published:** 2019-02-04

**Authors:** Nathan D. McDonald, Abish Regmi, Daniel P. Morreale, Joseph D. Borowski, E. Fidelma Boyd

**Affiliations:** Department of Biological Sciences, University of Delaware, 328 Wolf Hall, Newark, DE 19716 USA

**Keywords:** CRISPR-Cas systems, *Vibrio* species, Mobile genetic elements, Transposons, Tn7, Genomic islands, Horizontal gene transfer

## Abstract

**Background:**

Bacteria are prey for many viruses that hijack the bacterial cell in order to propagate, which can result in bacterial cell lysis and death. Bacteria have developed diverse strategies to counteract virus predation, one of which is the clustered regularly interspaced short palindromic repeat (CRISPR) and CRISPR associated (Cas) proteins immune defense system. Species within the bacterial family *Vibrionaceae* are marine organisms that encounter large numbers of phages. Our goal was to determine the significance of CRISPR-Cas systems as a mechanism of defense in this group by investigating their prevalence, phylogenetic distribution, and genome context.

**Results:**

Herein, we describe all the CRISPR-Cas system types and their distribution within the family *Vibrionaceae*. In *Vibrio cholerae* genomes, we identified multiple variant type I-F systems, which were also present in 41 additional species. In a large number of *Vibrio* species, we identified a mini type I-F system comprised of *tniQcas5cas7cas6f*, which was always associated with Tn7-like transposons. The Tn7-like elements, in addition to the CRISPR-Cas system, also contained additional cargo genes such as restriction modification systems and type three secretion systems. A putative hybrid CRISPR-Cas system was identified containing type III-B genes followed by a type I-F *cas6f* and a type I-F CRISPR that was associated with a prophage in *V. cholerae* and *V. metoecus* strains. Our analysis identified CRISPR-Cas types I-C, I-E, I-F, II-B, III-A, III-B, III-D, and the rare type IV systems as well as *cas* loci architectural variants among 70 species. All systems described contained a CRISPR array that ranged in size from 3 to 179 spacers. The systems identified were present predominantly within mobile genetic elements (MGEs) such as genomic islands, plasmids, and transposon-like elements. Phylogenetic analysis of Cas proteins indicated that the CRISPR-Cas systems were acquired by horizontal gene transfer.

**Conclusions:**

Our data show that CRISPR-Cas systems are phylogenetically widespread but sporadic in occurrence, actively evolving, and present on MGEs within *Vibrionaceae*.

**Electronic supplementary material:**

The online version of this article (10.1186/s12864-019-5439-1) contains supplementary material, which is available to authorized users.

## Background

Bacteriophages (phages) are viruses that infect bacteria, by injecting their viral DNA or RNA into bacterial host cells. This foreig'n DNA can then circularize and replicate or integrate into the bacterial host chromosome to form a prophage by site specific recombination mediated by an integrase. Phages are abundant in many ecosystems and are estimated to outnumber bacteria by ten-fold [1]. Some phages are useful to the bacterium by adding new genes and producing new phenotypes that can impact fitness and bacterial virulence [2–7]. However, many phages are harmful to their bacterial host causing bacterial cell lysis and death, and are important modulators of bacterial populations [8–10]. Bacteria have evolved mechanisms to protect against phage infection, including restriction modification (RM) systems and phage exclusion mechanisms—such as receptor modification. A more recent addition to this list is the **c**lustered **r**egularly **i**nterspaced **s**hort **p**alindromic **r**epeats (CRISPR) and **C**RISPR **as**sociated proteins (Cas) system [11–13]. This system is a bacterial immunity defense mechanism against foreign DNA such as phages and plasmids [13–17]. CRISPR-Cas systems are widespread among prokaryotes, found in ~ 84% of archaeal and ~ 47% of bacterial genomes [18]. A CRISPR-Cas system consists of three functional components: a set of *cas* genes, a leader sequence and a CRISPR array. The CRISPR array features direct repeats (DRs), which vary in size from 21 to 37-bp and can occur in 1 to over 100 times depending on the species and the strain. These repeats are separated by non-repetitive DNA of similar size called spacers [12]. The spacer sequences were shown to be acquired from previously infecting phage and act as memory to protect against future infection [14, 19]*.* Cas proteins are encoded by the *cas* gene cluster in an operon, which is usually located upstream from a CRISPR array. These Cas proteins are required for expression of *cas* genes, new spacer acquisition and target recognition and degradation [16, 20–24].

CRISPR-Cas systems are classified based on Cas protein content and arrangements in CRISPR-Cas loci with two main classes (1 and 2) and at least six types (I, II, III, IV, V, VI) that have been defined and updated [15, 25, 26]. The first level of classification is into class 1 and class 2, which separates CRISPR systems based on the type of CRISPR RNA (crRNA)–effector protein complexes that are utilized. Class 1 systems (type I, type III, and type IV) use a multi-subunit crRNA–effector complex, which is made up of several Cas proteins bound together with the mature crRNA to form a large protein complex. The function of this complex is to bind to the crRNA, which acts as a guide to target complementary foreign DNA and use its nuclease ability to cleave the targeted sequences [27, 28]. The class 2 systems consisting of type II, type V, and type VI have only one protein, Cas9, Cas12, and Cas13 respectively, that fulfills all the functions of the multiprotein effector complex [29].

There are currently six types of CRISPR systems characterized, which are differentiated based on their signature *cas* genes, gene arrangement and direct repeats. Signature Cas proteins are the effectors that cleave target DNA/RNA and are used to differentiate between these types. These hallmark proteins are Cas3 for type I, Cas9 for type II, Cas10 for type III, Csf1 for type IV, Cas12 (Cpf1) for type V, and Cas13 for type VI [15, 25, 26]. Among the system types, are subtypes that have additional signature genes and gene arrangements [15, 26]. These subtypes include: seven type I subtypes (I-A, I-B, I-C, I-U, I-D, I-E, and I-F), three type II subtypes (II-A, II-B, and II-C), four type III subtypes (III-A, III-B, III-C, and III-D), six type V subtypes (V-A, V-B, V-C, V-D, V-E, V-U), and three type VI subtypes (VI-A, VI-B, VI-C) [15, 26]. Common to almost all types of CRISPR systems is the presence of Cas1 and Cas2 proteins, which function in adaptation to add new spacer sequences into the CRISPR array [23]. However, recent studies indicate that these proteins are absent from a few active CRISPR-Cas systems*,* in which case the system is dependent on adaptation modules from other systems [15, 16, 23]. The most conserved CRISPR-associated proteins are Cas1 and Cas3 but in general Cas proteins are numerous and highly divergent making classification challenging [15, 25, 26, 30].

Spacers are sequences derived from protospacers from foreign invading mobile genetic elements (MGEs) that are incorporated into a CRISPR array between two repeat sequences generally adjacent to the CRISPR leader sequence. The protospacer adjacent motif (PAM) is a sequence consisting of two or three nucleotides immediately before the protospacer sequence and it is necessary to distinguish CRISPR targeted protospacer sequences (non-self) from the system’s own genome (self) [31].

The family *Vibrionaceae* have 8 genera that have whole genome sequences available, *Aliivibrio, Enterovibrio, Grimontia, Listonella, Vibrio, Photobacterium, Photococcus, Salinivibrio*) that are ubiquitous in the marine environment where phages are abundant. There have been limited studies describing CRISPR-Cas systems in this bacterial family and most studies were confined to *V. cholerae,* a significant pathogen of humans that causes cholera [15, 32–37]. A type I-F CRISPR-Cas system was described within a phage named ICP phages (International Centre for Diarrheal Disease Research, Bangladesh cholera phage), which was isolated from cholera stool samples [33]. A CRISPR-Cas type I-E system was described in *V. cholerae* biotype classical strains that caused the earlier pandemics of cholera [32, 34]. The type I-E system was present within a 17-kb genomic island (GI) named GI-24 [32]. GIs are non-self-mobilizing integrative and excisive elements that can contain a diverse range of traits and are present in a subset of strains of a species and absent from others. All GIs contain a recombination module comprised of an integrase required for site specific recombination, associated attachment sites (attL and attR) and, in many cases, a recombination directionality factor [38, 39]. GI-24 contained a recombination module and inserted between homologues of ORFs VC0289-VC0290 in the El Tor strain N16961, which lacked this island [32, 40]. Recently, a type I-F CRISPR-Cas system was identified within a 29-kb genomic island named Vibrio Pathogenicity Island-6 (VPI-6) that contained a recombination module and could excise from the chromosome as a complete unit [36]. Thus to date, the CRISPR-Cas systems that have been identified in *V. cholerae* are all present within MGEs.

Here, we determined the prevalence, diversity and phylogenetic distribution of CRISPR-Cas systems present within *Vibrionaceae* through comparative genomics, bioinformatics, and phylogenetic analyses of available genome sequences in the NCBI database. In addition, we examined the genomic context of each system to determine whether it was acquired as a single module or within a MGE. Several variant type I-F CRISPR-Cas systems were identified in *V. cholerae* and in 41 additional species. The canonical type I-F system and a variant type I-Fv in *V. cholerae* were present within the genomic island VPI-6. A mini type I-F system (*tniQcas5cas7cas6f*) was within Tn7-like transposons in *V. cholerae, V. parahaemolyticus* and over 40 additional species. In *V. parahaemolyticus,* within the Tn7-associated CRISPR-Cas system was the pathogenicity island containing the type three secretion system 2 (T3SS-2). A putative hybrid type III-B/I-F system, which contained a type III-B *cas* gene cluster, a *cas6f* gene and a type I-F CRISPR array was identified in several *V. cholerae* and *V. metoecus* strains. The hybrid system was present within a prophage at the same genome location in both species. Multiple CRISPR-Cas system types including type I-C, I-E, I-F, II-B, III-A, III-B, III-D and the rare type IV system were uncovered. Interestingly, the majority of these CRISPR-Cas systems were identified within MGEs that included genomic islands, plasmids and transposon-like elements. A number of novel *cas* gene arrangements and *cas* gene contents were found among the systems identified. The data suggest that many variations of Cas protein content exist within the different types, and the acquisition of CRISPR-Cas systems on MGEs is a common feature in this group. Phylogenetic analysis of Cas proteins and their sporadic occurrence within a species also suggested that the CRISPR-Cas systems were acquired by horizontal gene transfer. Overall these data show that CRISPR-Cas systems are phylogenetically widespread but not the predominant defense mechanism of this group.

## Results

### CRISPR-Cas systems present in the family *Vibrionaceae*

Using BLAST and comparative genome analyses, we examined species belonging to the family *Vibrionaceae* available in the NCBI genome database for the presence of CRISPR-Cas systems. We identified eight different system types: type I-C, I-E, I-F, II-B, III-A, III-B, III-D, and IV as well as variants of these types and hybrid systems among 70 species (Additional file 1: Figure S1A). These CRISPR-Cas systems were sporadic in their occurrence and distribution within and among species. The majority of the systems were detected on MGEs such as genomic islands, plasmids, and transposon-like elements suggesting a possible vector for horizontal gene transfer (Additional file 1: Figure S1B). The most predominant type identified was the type I system, which accounted for 81% of the systems identified that encompassed type I-F, type I-E and type I-C systems. Within the type I systems, the type I-F subtype was the most abundant and was found across four genera, 41 species, and 116 strains (Additional file 1: Fig. S1C). A type II-B system was present in two *Vibrio* species and three *Salinivibrio* species (Additional file 1: Figure S1C). The type III systems were the next most prominent type making up 14% of the systems identified consisting of type III-A, type III-B, and type III-D. The rare type IV system was identified on a plasmid in two strains of *V. parahaemolyticus*. The distribution data, only present in a few strains of a particular species, leads to the most parsimonious conclusion that CRISPR-Cas systems are not ancestral to any species within this family.

### CRISPR-Cas type I-F systems in *V. cholerae*

**Fig. 1:**
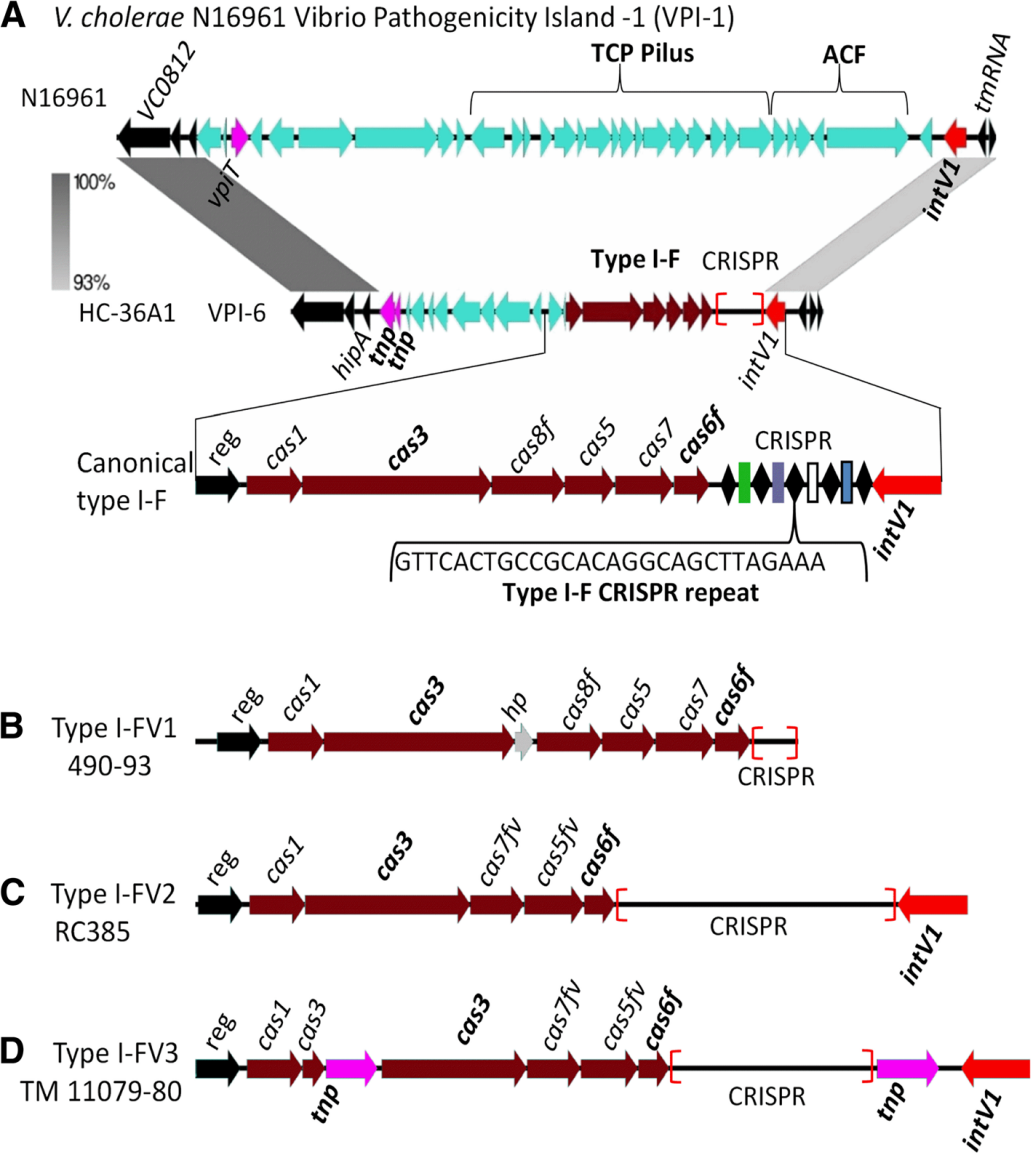
CRISPR-Cas type I-F systems in *Vibrio cholerae*. **a** A canonical type I-F system present within VPI-6 in *V. cholerae* strain HC36A1 at the same location as VPI-1 in N16961. **b** Variant type I-FV1 in strain 490–93 contains one additional gene encoding a hypothetical protein (HP). **c** Type I-FV2 in strain RC385 lacks a *cas8* gene and contains variant *cas7fv* and *cas5fv* genes. **d** Type I-FV3 in strain TM 11079–80 contains a transposase (*tnp*) inserted in *cas3*, no *cas8* homolog is present and contains the variant *cas7fv* and *cas5fv* genes. Genomic comparisons and gene arrangements were constructed via Easyfig [74]. Grayscale bars represents nucleotide homology. Black diamonds of CRISPRs represent direct repeats and colored rectangles represent spacers. The core genes are colored in black, transposases in pink and integrases in red. Red brackets indicate the genomic location of CRISPR

Using the Cas1, Cas3 and Cas6f proteins from the previously identified type I-F system in *V. cholerae* HC-36A1 as seeds, we examined *V. cholerae* genomes in the NCBI genome database using BLAST analysis. This analysis revealed the presence of variants of the type I-F system in addition to the canonical system (Fig. 1). A total of 35 distinct non-pathogenic *V. cholerae* strains contained a type I-F system (Additional file 2: Table S1), with 16 strains containing a canonical type I-F system consisting of six *cas* genes (*cas1cas3cas8fcas5cas7cas6f)* followed by a type I-F CRISPR array **(**Fig. 1a). One strain 490–93 had an additional gene between *cas3* and *cas8f* that encodes a hypothetical protein (Fig. 1b). A type I-F system comprised of five *cas* genes (*cas1cas3cas7fvcas5fvcas6f*) was present in 13 strains, which encodes variant Cas7fv and Cas5fv proteins (Fig. 1c). This system shared homology with the type I-Fv previously described in *Shewanella putrefaciens* strain CN-32 that was shown to be an active system [41]. One strain, TM 11079–80, contained a CRISPR-Cas system comprised of *cas1cas3tnpcas3cas7fvcas5fvcas6f* with the *cas3* gene and the CRISPR array flanked by transposase genes (Fig. 1d). These type I-F systems were all present in non-choleragenic strains within the previously described region VPI-6, which has all the hallmarks of a GIs acquired by horizontal gene transfer (HGT) [38, 39, 42]. VPI-6 is a ~ 29-kb island with a %GC content lower than the overall GC content of the genome and is absent from *V. cholerae* N16961, the cholera pandemic strain [36]. The island contains an integrase from the tyrosine recombinase family, which mediates site specific integration and excision of the island from the genome [36, 42, 43]. The ends of the islands are flanked by attachment sites, *attL* and *attR* marking the insertion site [36, 42]. Previously, we demonstrated that VPI-6 can excise from the chromosome as a complete unit indicating that the CRISPR-Cas system can be transferred with the rest of the island [36].

To determine whether the CRISPR-Cas system and the VPI-6 island had a similar evolutionary history and were acquired together, phylogenetic analysis of the *cas1* gene from the type I-F system and *intV* gene from VPI-6 was performed. Overall there was congruency between the *intV* and *cas1* gene trees, with four divergent branches within the *intV* tree, and strains found within these four divergent branches showed a somewhat similar branching pattern in the *cas1* gene tree. This would suggest a similar evolutionary history. A few strains, 2012Env-2, HE-48, A325, showed different clustering patterns between the trees. However, the bootstrap values for many branches for the *cas1* tree were low indicating the branching patterns are not robust as there were limited polymorphic sites (Additional file 1: Figure S2).

### A mini type I-F system within a Tn7-like transposon

**Fig. 2:**
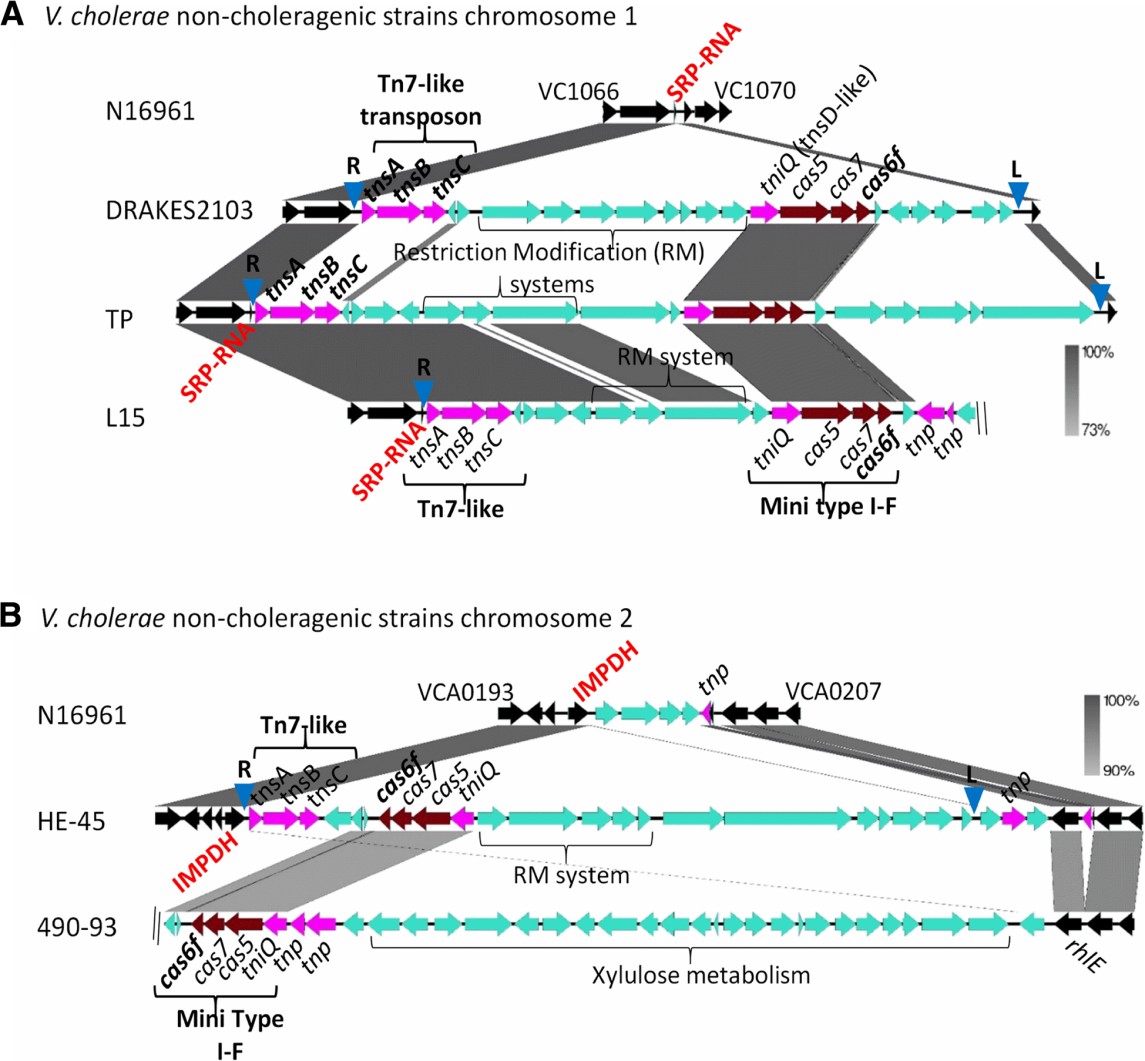
Tn7-like transposons carrying CRISPR-Cas systems in *V. cholerae.* The Tn7-like transposon associated mini type I-F variant systems and their cargo genes identified in *V. cholerae* strains were constructed and compared using EasyFig. **a** *V. cholerae* strains DRAKES2103, TP and L15 with Tn7-like elements at the SRP-RNA locus. **b** *V. cholerae* strain HE-45 with Tn7-like element at the IMPDH locus. Grayscale bars represents nucleotide homology. The core chromosomal genes are colored in black, Tn7-like transposon and other transposase genes are in pink. Blue inverted triangles represent right end and left end attTn7 attachment sites

Our analysis identified five *V. cholerae* strains that contained a type I-F CRISPR-Cas region comprised of a four gene cluster, *tniQcas5cas7cas6f* (Fig. 2; Additional file 2: Table S1). In this system, *cas5* is a fusion between *cas8f* and *cas5f* and lacked the adaptation proteins Cas1 and Cas2 as well as Cas3 required for target cleavage. The system was homologous to a previously described Cas1-less minimal system associated with a Tn7-like transposon in a large number of bacterial species [44]. The canonical Tn7 transposon is comprised of five genes *tnsABCDE*, which encode a TnsAB transposase, a regulator TnsC, and TnsD and TnsE required for target site insertion at a Tn7 specific attachment generating right end (R) and left end (L) att sites [45, 46]. In *V. cholerae* strains, the Tn7-like region consists of four genes, *tnsABC* homologs in an operon, and *tniQ* in an operon with *cas5cas7cas6f* genes. TniQ shows homology to TnsD, which targets a sequence specific site, attTn7, for insertion. No *tnsE* homolog was identified, which is usually responsible for directing the element into other MGEs [47, 48]. The CRISPR array associated with this system was short, containing two to three spacers and a type I-F direct repeat.

Examination of the genome context of these mini type I-F systems in *V. cholerae* strains identified the presence of three divergent mini type I-F systems. These mini type I-F systems were associated with at least three divergent Tn7-like transposons (Fig. 2a). In *V. cholerae* strains DRAKES2103, a 27-kb region was identified that was absent from N16961. The region encompassed the Tn7-like transposon, the mini type I-F CRISPR-Cas system and also contained a restriction modification system (Fig. 2a). To determine whether the entire region was cargo within the Tn7-like transposon, we examined the insertion site for Tn7-like right and left end attachment sites. In *V. cholerae* DRAKES2103, the element is inserted downstream of the gene for signal-recognition particle RNA (SRP-RNA), a novel Tn7 transposon attachment sites that was described previously by Peters and colleagues [44]. By convention the insertion site closest to *tnsA* is called the right (R) end and the distal end is named the left (L) end (Fig. 2a). Transposition generates a direct repeat usually 5-bps at each end, followed by a 3-bp sequence TGT and a 22-bp TnsB binding site at the right end, and at the left end a 3-bp ACA preceding the direct repeat which is itself preceded by a TnsB binding site (Table 1). In DRAKES2103, we identified the R and L end attTn7 sites that flanked the entire region suggesting mobilization by the element (Fig. 2a; Table 1). A divergent Tn7-like transposon was identified in *V. cholerae* TP, which encompassed a 35-kb element that included the mini type I-F system and a restriction modification system. We were also able to detect the R and L site flanking the entire region (Fig. 2; Table 1). Similarly, in strain L15, the element was inserted at the same site, however, the contig was short and the left end could not be determined (Fig. 2a).

**Table 1:** Mini type I-F-carrying Tn7 R-end and L end attachment sites

**Species/strain**	**Insertion locus**	**Right end (R)**	**Left end (L)**	**Tn7 size**	**Cargo genes**
***V. cholerae***					
DRAKES2103	SRP-RNA	**atctg**tgtcgctgaaagcataaagtgtccaattta	cgcataggacatcttatgctttcagcgaca**atctg**	27-kb	RM1 system
TP	SRP-RNA	**tctga**tgtttgcaaaataagttcgcataaattgca	gctatgcagacttatgctgcaagcatcaca**tctga**	35-kb	RM1 system
L15	SRP-RNA	**tctga**tgtttgcaaaataagttcgcataaattgca		Short contig	RM2 system
HE-45	IMPDH	**acatt**tgttgatacaaccataaaatgataattaca	attaaatatcactttatggttgcatcaaca**acatt**	36-kb	RM3 system
***V. parahaemolyticus***					
RIMD2210633	*yciA*	**gagtt**tgtaaatacaaccatacattgcaacaatac	tataaatgtcactttatggttgtatcaaca**gagtt**	80-kb	T3SS-2α
BB22OP	*yciA*	**gagtt**tgtaaatacaaccatacattgcaacaatac	tataaatgtcactttatggttgtatcaaca**gagtt**	80-kb	T3SS-2α
TH3996	*yciA*	**gagtt**tgtaaatacaaccatacattgcaacaatac	tataaatgtcactttatggttgtatcaaca**gagtt**	98-kb	T3SS-2β
MAVP-Q	*yciA*	**tgagt**tgttgatacaaccataaaatgataattaca	tataaatatcactttatggttgtatcaaca**tgagt**	107-kb	T3SS-2γ
ISF-25-6	IMPDH	**aataa**tgttgaaacaaccataaattgatatttaca	tacaattatcaatttatggttgtttcaaca**aataa**	35-kb	RM4 system
UCM-V493	SRP-RNA	**cttta**tgaagcctgcaatatatgttcgcataaatt	ggactatgctaaattacgttgcaggcatca**cttta**	20-kb	
CFSAN007439	SRP-RNA	**cttta**tgaagcctgcaatatatgttcgcataaatt	ggactatgctaaattacgttgcaggcatca**cttta**	23-kb	
CDC_K4762		**cttta**tgaagcctgcaatatatgttcgcataaatt		Short contig	TA system
***V. navarrensis***					
ATCC 51183	SRP-RNA	**gagct**tgaagaacgatgctgttgttcgcactctct	tgtggcgaccaacttgccacaacccggtca**gagct**	51-kb	RM system Type I-C CRISPR-Cas

In *V. cholerae* HE-45, the Tn7-like transposon was inserted at another novel Tn7 insertion site downstream of inosine-5′-monophosphate dehydrogenase (IMPDH) also annotated as guanosine 5′-monophosphate oxidoreductase (*guaC*) (Fig. 2b) (Table 1). The Tn7-like element encompassed a 36-kb region, containing a mini type I-F system and a restriction modification system. In strain 490–93, at the same genomic location in which the Tn7-like element for HE-45 was inserted, we identified a region with a mini type I-F system and a xylulose metabolism gene cluster, however due to short contig, we were unable to locate a Tn7-like element or R and L sites (Fig. 2b). This is the only *V. cholerae* strain in the NCBI genome database (> 900 genomes sequenced) that contains this metabolic cluster. Overall it appears that these Tn7-like elements have captured not only CRISPR-Cas defense systems but also restriction modification systems.

**Fig. 3:**
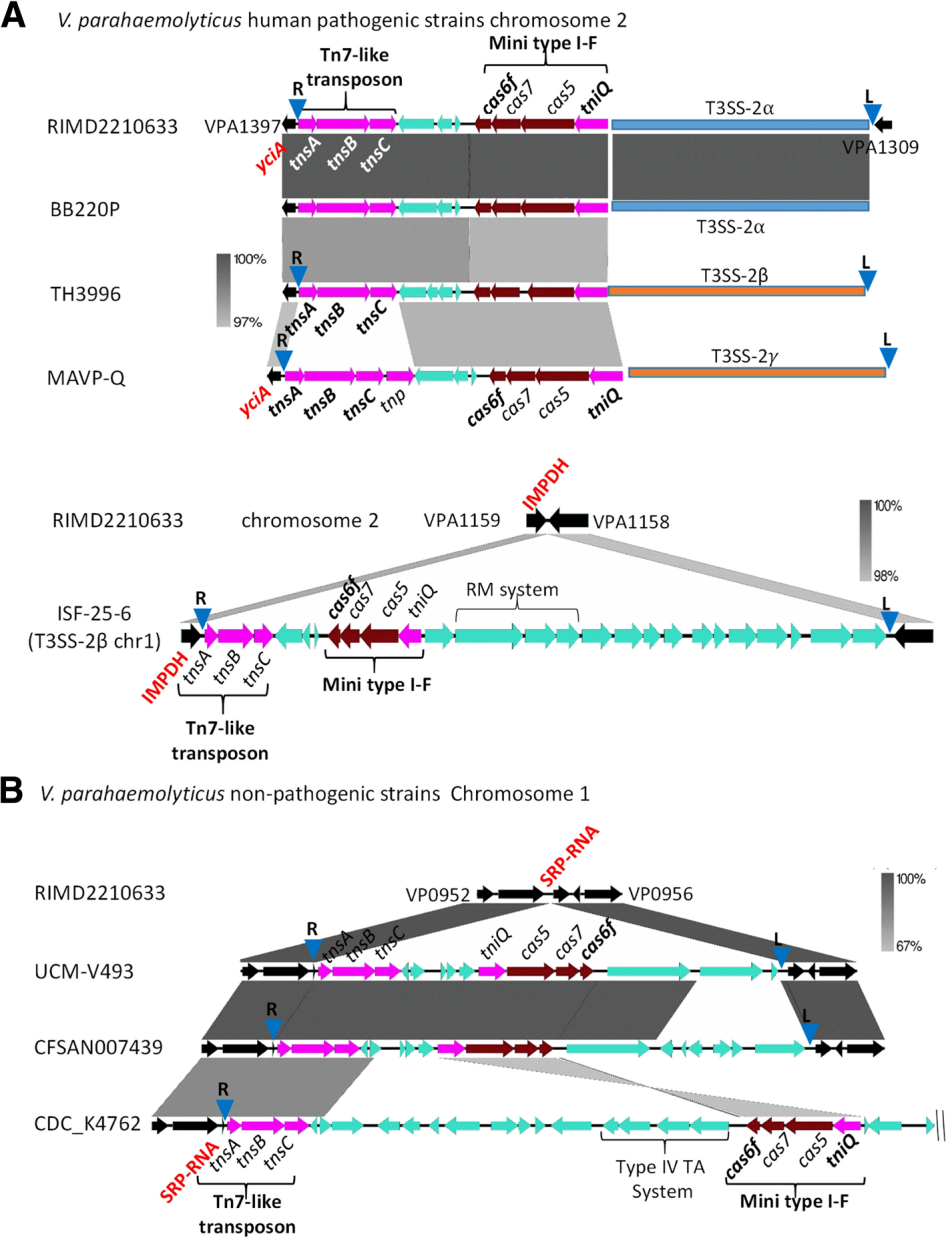
Tn7-like transposons carrying CRISPR-Cas systems in *V. parahaemolyticus.* **a** The Tn7-like associated mini type I-F system region in representative *V. parahaemolyticus* strains RIMD2210633, BB22OP, TH3996, and MAVP-Q present on chromosome 2 at the *yciA* locus insertion site and the Tn7-like associated mini type I-F system region in representative strain ISF-25-6. **b** The Tn7-like associated mini type I-F system region in representative strains UCM-V493, CFSAN007439 and CDC_K4762 within chromosome 1 at the SRP-RNA insertion site. Grayscale bars represents nucleotide homology. The core chromosomal genes are colored in black, Tn7-like transposon and other transposase genes are in pink. Blue inverted triangles represent right end and left end attTn7 attachment sites

BLAST analysis using TniQ identified at least 40 additional species from the family *Vibrionaceae* that contained a copy of this protein and an associated Cas6f. Interestingly, a large number of *V. parahaemolyticus* strains (> 800 sequenced genomes) in the NCBI genome database contained a Tn7-like associated mini type I-F system. In this species, four divergent mini type I-F systems were identified that were carried within five different Tn7-like transposons, which had three different attachment sites; SRP-RNA on chromosome 1, and IMPDH and YciA (acyl-CoA thioester hydrolase) on chromosome 2 (Fig. 3). In all *V. parahaemolyticus* strains that contained a type three secretion system-2α (T3SS-2 α) on chromosome 2, the Tn7-like CRISPR-Cas region was directly upstream of the T3SS-2α gene cluster at the *yciA* R end attachment site (Fig. 3a). T3SS-2α is a contact dependent secretion system that delivers effector proteins directly into target eukaryotic cells and is the primary virulence mechanism of pandemic *V. parahaemolyticus* strains [49–51]. T3SS-2α was previously shown to be present on an 80-kb pathogenicity island named VpaI-7 that is only present in pathogenic isolates [49, 52, 53]. To investigate whether T3SS-2α is within the Tn7-like element, we examined the region for an attTn7 L end site and identified the L end at the 3′ end of the island (Fig. 3a). *Vibrio parahaemolyticus* strains that contained the non-homologous T3SS-2β system also contained an associated mini type I-F CRISPR-Cas system at the Tn7-like insertion site *yciA* (Fig. 3a). This entire region was flanked by R end and L end attTn7 sites (Fig. 3a and Table 1). In strain MAVP-Q, a variant of the T3SS-2β gene cluster named T3SS-2α is also present at this site but contains a highly divergent Tn7-like system with unique attTn7 sites flanking the 107-kb region. These data suggest a possible mechanism of mobilizing T3SS-2 within this species.

In strain ISF-25-6 that contains a T3SS-2β on chromosome 1, a variant Tn7 associated mini CRISPR-Cas type I-F system was present on chromosome 2 at the IMPDH locus between VPA1158 and VPA1159 relative to strain RIMD2210633 (Fig. 3a) (Table 1). This region also contains a restriction modification system and the entire 35-kb region is flanked by attTn7 sites (Table 1). In non-human pathogenic strains, the Tn7 associated mini type I-F system is located in chromosome 1 at the SRP-RNA insertion site between VP0953 and VP0954 relative to RIMD2210633 (Fig. 3b). At this site depending on the strain, two divergent CRISPR-Cas systems were present associated with two divergent Tn7-like transposons. In one strain CDC_K4762, the region also contained a type IV toxin antitoxin system, we were able to identify the R site, however due to a short contig, the L site could not be determined (Fig. 3b) (Table 1). Comparative genomic analysis indicated that the Tn7 associated CRISPR-Cas mini type I-F was acquired at least four times in this species.

**Fig. 4:**
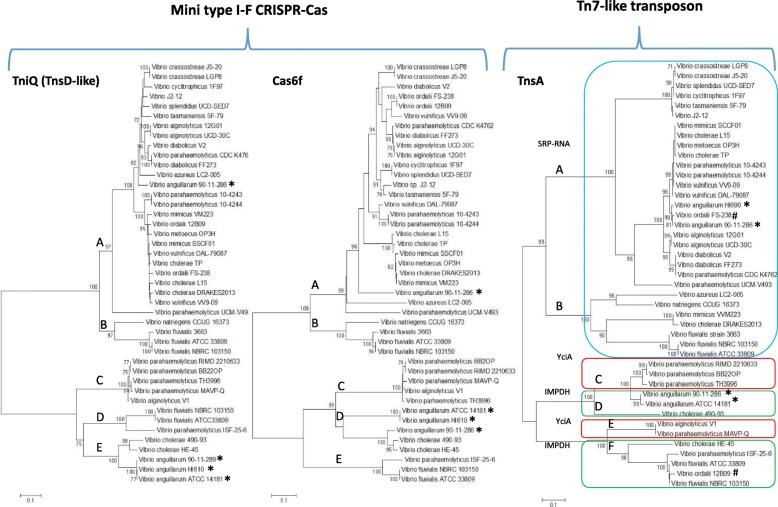
Phylogenetic analysis of TniQ (TnsD-like), Cas6f and TnsA among *Vibrionaceae*. A total of 43 sequences for each protein was examined representing the mini type I-F system and the associated Tn7-like transposon. Sequences for UCM-V493 also represent those for CFSAN007439. Protein sequences were aligned in MEGA7 using CLUSTALW. Sequences were used to construct a neighbor-joining tree with bootstrap of 1000

To investigate further the evolutionary history of the Tn7-like transposons and the mini type I-F systems, we performed phylogenetic analysis on a select number of strains and species for which we knew that TniQ (TnsD-like), Cas6, and TnsA were co-located on the same contig (Fig. 4). For the TniQ and Cas6f trees, at least five highly divergent clades are present named A, B, C, D, and E and within each clade are several divergent lineages. For the most part, the TniQ and Cas6f trees showed overall congruency suggesting a shared evolutionary history, with a few exceptions. For the TniQ and Cas6f proteins present in *V. cholerae*, three strains cluster together in clade A and two strains clustered together within clade E in both trees. Similarly, pathogenic strains of *V. parahaemolyticus* clustered together within clade C whereas nonpathogenic strains cluster mainly within clade A on both trees. In both trees, TniQ and Cas6f from *V. anguillarum* and *V. fluvalis* clustered within clades D and E (Fig. 4). These data indicate both proteins share a similar evolutionary history in these species. In strains of both *V. anguillarum* and *V. fluvalis*, two non-homologous mini type I-F systems were present, and the second system is present at the SRP-RNA locus attTn7 site within clade A and B, respectively. Similarly, in the TnsA tree, for the most part, the clustering patterns hold, indicating that both the CRISPR systems and Tn7-like transposons share a similar evolutionary history suggesting they were acquired together as an evolutionary unit. However, some key discrepancies are present between the CRISPR protein trees and the Tn7 TnsA tree in a few species (Fig. 4). For example, *V. anguillarum* strain HI610, which clusters with *V. anguillarum* ATCC14181 in the TniQ and Cas6f trees is present on a divergent branch in clade A on the TnsA tree. Similarly, two *V. ordallii* strains cluster together on the CRISPR protein trees but are present on two divergent clades within the TnsA tree. This indicates two different Tn7-like transposons present at different attachment sites suggesting recent horizontal transfer (Fig. 4). This is also found for *V. cholerae* DRAKES2013 and *V. mimicus* VVM223, whose CRISPR region cluster together with other *V. cholerae* and *V. mimicus* strains but contained two divergent Tn7-like transposons but in this case, they are present at the same chromosomal insertion site (Fig. 4).

### Putative hybrid CRISPR-Cas type III-B/I-F system

**Fig. 5:**
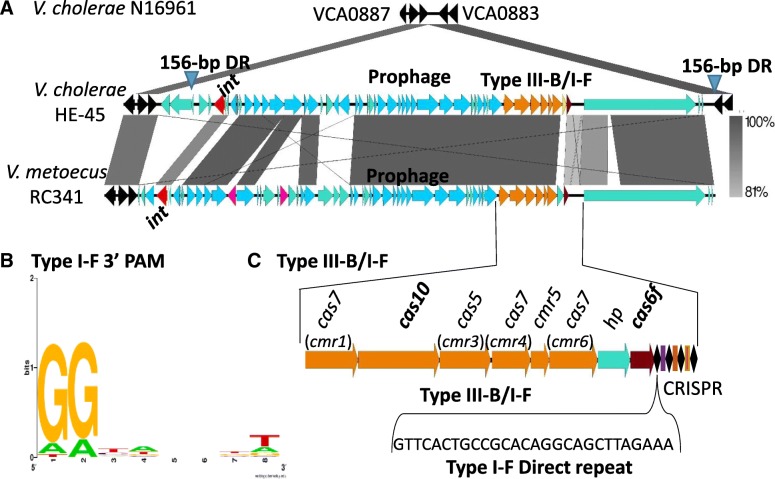
Hybrid CRISPR-Cas type III-B/I-F system associated with a prophage in *V. cholerae* and *V. metoecus.* **a** Genome comparison of *V. cholerae* N16961 and HE-45. The hybrid system is at the 3′ region of a prophage located between VCA0884 and VCA0885 relative to the reference strain N16961, which lacks the region. A homologous phage was found in *V. metoecus* RC341 and was inserted at the same genomic location. **b** The conserved protospacer adjacent motif (PAM) was identified by analyzing the protospacers and is represented using Weblogo. **c** The hybrid system consists of type III-B *cas7cas10cas5cas7cmr5cas7* followed by a resolvase and *cas6f* and a type I-F CRISPR. The core genes are colored in black, putative phage genes are colored in light blue, putative integrases are colored in red. Genomic comparisons and gene arrangements were constructed in Easyfig. Grayscale bars represent nucleotide homology. Inverted triangles represent a 156-bp direct repeat

We identified eight *V. cholerae* strains that contained a putative type III-B/I-F hybrid system that consisted of type III-B operon *cas7cas10cas5cas7cmr5cas7* followed by a resolvase and *cas6f* genes (Additional file 2: Table S7). One exception was strain BRV8, which had the same type III-B system genes followed by a gene encoding a hypothetical protein and then *cas6f*. The type III-B/I-F system in all 8 strains was associated with a 62-kb region inserted within chromosome 2 at ORF VCA0885 relative to the El Tor reference genome N16961, which lacked the region (Fig. 5a). Analysis of this 62-kb region using Phaster [54], a phage identification tool, showed that the region had homology to a *Shigella* phage Ss-VASDF (Fig. 5a). A 156-bp direct repeat was identified at the ends of the region suggesting the CRISPR-Cas system could be mobilized within the prophage (Fig. 5a).

The CRISPR array associated with the type III-B/I-F hybrid system contained a type I-F direct repeat and a type I-F PAM (Fig. 5c and b). In addition, in two *V. metoecus* strains YB4D01 and RC341, a highly similar type III-B/I-F hybrid system within a prophage highly homologous to that present in *V. cholerae* HE-45 was identified (Fig. 5a) (Additional file 2: Table S7). Phylogenetic analysis of an integrase gene, *cmr1,* and *cas6f* genes among five strains showed no congruency suggesting no shared evolutionary history. However, there was a limited number of polymorphic sites among the strains examined (Additional file 1: Figure S3).

Next, we examined the CRISPR arrays associated with the type I-F system and the putative type III-B/I-F hybrid system identified in *V. cholerae*. The CRISPRMap program classified the direct repeat sequence as a type I-F system repeat in all strains analyzed (Additional file 2: Tables S1 and S7). The arrays analyzed ranged in size from 2 to 83 spacers and a total of 1504 spacers were identified. Using the CRISPRTarget program to identify spacer homology in the plasmid and phage databases, we found that 356 of the 1504 spacers hit to protospacers. A total of 215 spacers matched to regions within the same sequences of phages X29/phi-2 (accession number KJ572845) and Kappa, as well as three filamentous phages fs2, fs-1 and KSF1 (Additional file 1: Figure S4). In *V. cholerae* strain 984–81, spacers had four targets to CTXphi. Several spacers were also found to target Vibrio phages, pYD21-A, YFJ, CP-T1, and Martha 12B12.

### CRISPR-Cas type I-F systems within mobile genetic elements (MGEs)

We determined that 97% of the type I-F systems identified in this study were associated with MGEs, which was based on the presence of signature genes in the vicinity of the CRISPR-Cas genes and comparative genome analysis. For example, the type I-F system in *V. metoecus* YB5B04 was present within an 18-kb island integrated between a gene encoding a hypothetical protein and *trmA,* with respect to *V. metoecus* OYP8G12, which lacked the island (Additional file 1: Figure S5A). The 5′ end of the island was marked with *int*, which encoded a putative integrase required for site specific recombination. We also identified *attL* and *attR* sites flanking the island indicating that the 18-kb region was likely acquired as a unit by site specific recombination (Additional file 1: Figure S5A). The GC content of this region was 43% compared to the overall genome GC content of 47% suggesting it is not ancestral to the genome. In *V. parahaemolyticus* A4EZ703, a type I-F system was present within an island inserted between VPA0712 and VPA0713 with respect to *V. parahaemolyticus* RIMD2210633 that lacked the region (Additional file 1: Figure S5B). The 63-kb island had a GC content of 41%, compared to 45% across the genome. This island contained an integrase at its 3′ end and the island was flanked by *attL* and *attR* sites (Additional file 1: Figure S5B). The type I-F system in *V. vulnificus* 93 U204 was present within a 25-kb genomic island inserted between VV1_0634 and the tRNA-Met locus with respect to *V. vulnificus* CMCP6 that lacked the region (Additional file 1: Figure S5C). The type I-F system was also present within a genomic island region in *V. fluvialis* that contained an integrase gene (Additional file 1: Figure S5D). Although the CRISPR-Cas system are identified within different genomic islands in these strains, it is not possible to determine whether they were acquired with the island or whether they are a recent addition to the island.

### Phylogenetic analysis of the Cas6f proteins

**Fig. 6:**
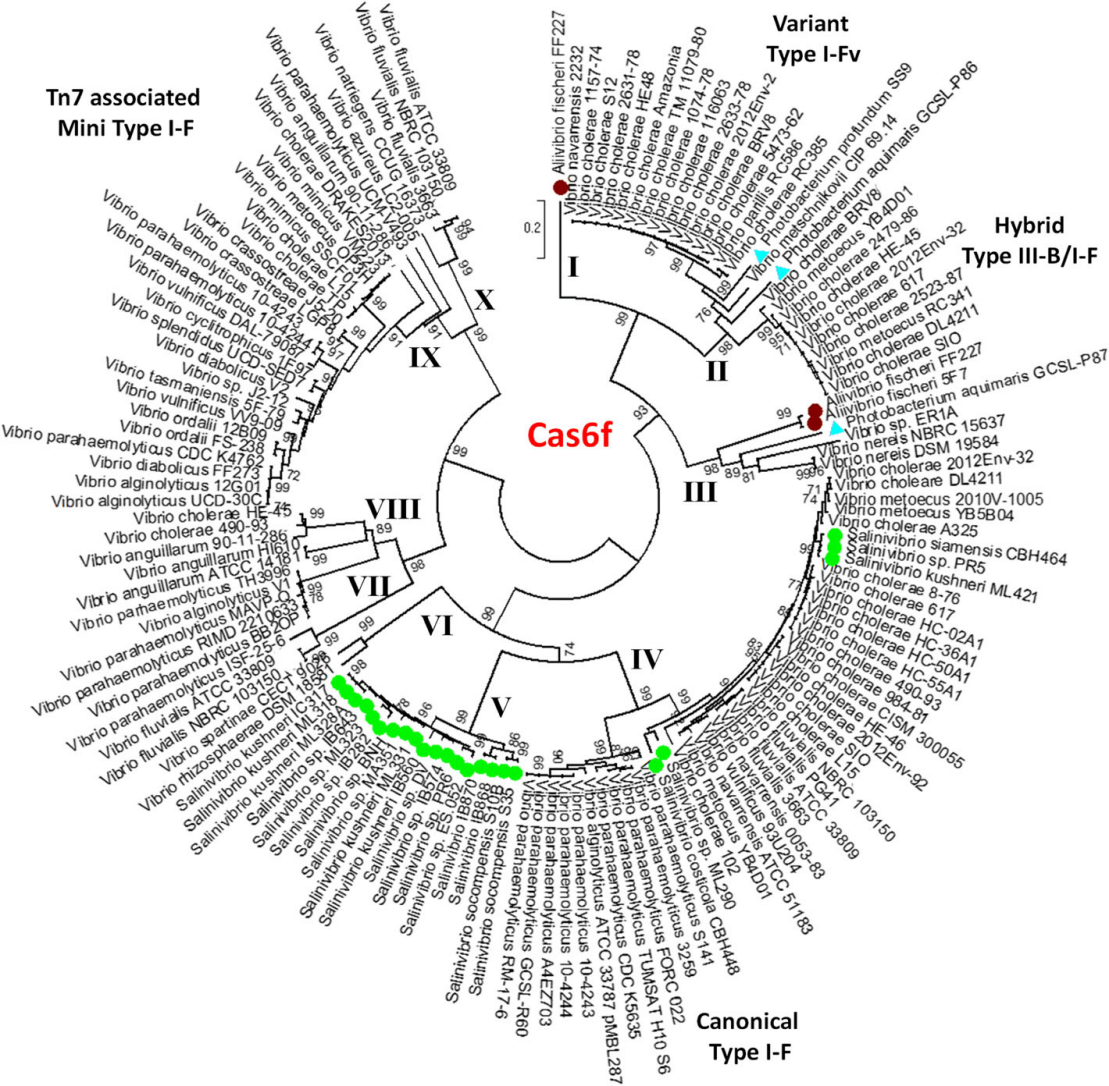
Phylogenetic analysis of Cas6f from *Vibrionaceae*. Cas6f protein sequences were examined from all characterized type I-F systems identified in the *Vibrionaceae*. Protein sequences were aligned in MEGA7 using CLUSTALW. Sequences were used to construct a neighbor-joining tree with bootstrap of 1000. Members of the *Salinivibrio* are marked with green circles, the *Photobacterium* are indicated by light blue triangles and the *Aliivibrio* are indicated by dark red circles

All Cas6f proteins identified in this study (Additional file 2: Tables S1, S2 and S7**)** were aligned using ClustalW and a phylogenetic tree was constructed by the neighbor-joining method [55, 56] (Fig. 6). The Cas6f proteins clustered into 10 major clades that had strong bootstrap values. The Cas6f proteins clustered together based on the type I-F system in which it was found. Clade I contained 13 *V. cholerae* strains and 1 *V. parillis* (formerly *V. cholerae*) strain, recovered between 1978 and 2009 from five continents and was associated with the variant type I-F (type I-FV2) systems containing five *cas* genes. Clade II contained Cas6f from 10 strains with the type III-B/I-F hybrid system, which were isolated mainly in the USA and Haiti with single strains from both the Ukraine and the UK. Clade III contained six strains, containing the canonical type I-F system from three genera, *Aliivibrio, Photobacterium* and *Vibrio*. Clade IV contained 17 *V. cholerae* strains that were recovered between 1981 and 2012 from four continents and was associated with the canonical type I-F system. Within this clade and closely related to *V. cholerae* were Cas6f from *V. metoecus, V. fluvialis, V. navarrensis* and five strains of *Salinivibrio*. (Fig. 6; Additional file 2: Table S1). *Salinivibrio* is a highly divergent species from *Vibrio* species in general [57]. Thus, this cluster within clade IV represents another example of horizontal transfer between two distantly related genera. Evidence of horizontal gene transfer include the presence of a Cas6f protein from *V. navarrensis* that was nested with *V. cholerae* Cas6f proteins within clade IV (Fig. 6). It was previously shown that *V. navarrensis* is distantly related to *V. cholerae* [58]. Clade IV also contains a lineage comprised of Cas6f from 10 *V. parahaemolyticus* strains, which all contained a canonical type I-F system, present within a genomic island at the same genome location that varied in size from 75-kb to 135-kb depending on the strain. Clade V consists of Cas6f proteins entirely from *Salinivibrio* species and was distantly related to those present in other *Vibrionaceae* genera (Fig. 6). Clade VI contained Cas6f from two *Vibrio* species, *V. rhizosphaerae* and *V. spartinae* (Fig. 6).

The Cas6f associated with the Tn7-like transposon mini type I-F system formed four highly divergent branches (clade VII-clade X) within which were highly variant Cas6f proteins, with some species present on multiple distantly related branches indicating in some species the system was acquired multiple times from diverse sources (Fig. 6).

### Type I-E CRISPR-Cas systems in *V. cholerae*

**Fig. 7:**
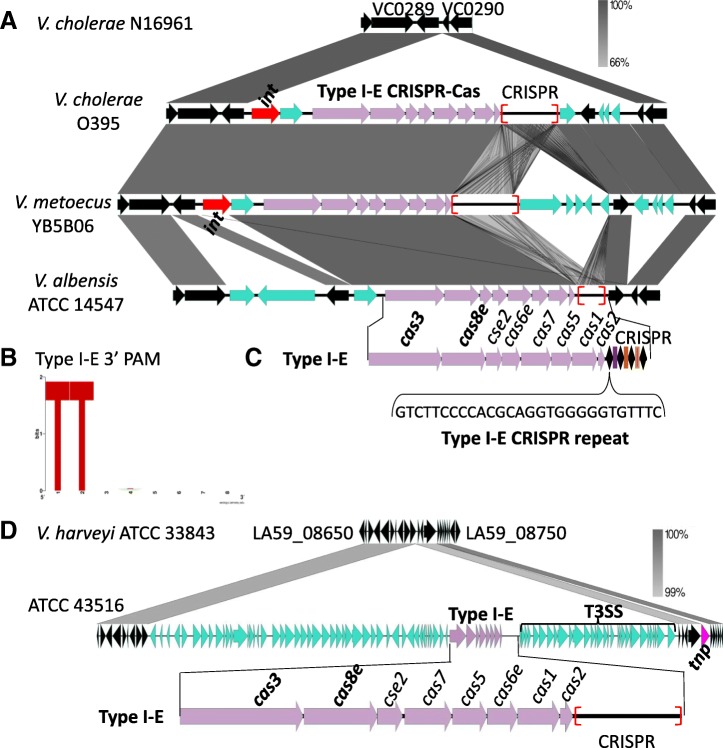
CRISPR-Cas type I-E system present on GI-24. **a** Type I-E CRISPR-Cas island characterized in classical *V. cholerae* strains (O395) inserted between VC0289 and VC0290 relative to the El Tor biotype strain N16961 that lacked the island. **b** These type I-E systems have a conserved PAM of 3’ NTT 5′ and **c** a canonical type I-E direct repeat. Genomic comparisons were performed using Easyfig and grayscale bars represent nucleotide homology. The core genes are colored in black, transposases are colored in pink and integrases are red. Red brackets indicate the genomic location of CRISPR. **(d)** CRISPR-Cas island in *V. harveyi* ATCC 43516, which also contains a type three secretion system (T3SS) and is inserted between LA59_08695 and LA59_08700 relative to *V. harveyi* ATCC 33843 that lacks the region

A total of 29 *V. cholerae* strains contained a type I-E system (18 strains were previously described as classical biotype *V. cholerae* strains) (Additional file 2: Table S3) [34, 40]. In all strains, the type I-E system was carried on the genomic island GI-24 that is absent from N16961 (Fig. 7a) [34, 40]. It was demonstrated that the type I-E system present in the classical biotype strains was functional [34]. All strains contained the *cas3cas8ecse2****cas6cas7cas5****cas1cas2* gene cluster followed by a CRISPR array and a canonical type I-E repeat (Fig. 7c). This is a variant *cas* gene arrangement for type I-E systems as the canonical *cas* gene arrangement is described as *cas3cas8ecse2****cas7cas5cas6****cas1cas2* [15]. CRISPR arrays in these strains contained between 2 and 80 spacers and 44 of the 330 spacers targeted the Vibrio phage X29/phi2. Analysis of the protospacers identified allowed us to determine the PAM of these systems which we found to be 3’ NTT 5′ as previously describe for type I-E systems (Fig. 7b) [34].

### Type I-E CRISPR-Cas systems in *Vibrionaceae*

A total of 28 strains encompassing ten species of *Vibrio*, four species of *Photobacterium*, nine species of *Salinivibrio* contained a type I-E system (Additional file 2: Table S4). In *V. metoecus* YB5B06, the type I-E system was present within GI-24 similar to *V. cholerae* classical strains (Fig. 7a). The type I-E system was also identified in *V. albensis* strains ATCC 14547 and VL426 and was present in a 12-kb genomic region inserted at the same genomic location as GI-24 with respect to N16961, however, no integrase was identified (Fig. 7a; Additional file 2: Table S4).

*Vibrio azureus* LC2–005 and NBRC 104587 also contained the I-E system, each with two CRISPR arrays. We did not identify any spacer hits for either of these two strains. A canonical type I-E system consisting of *cas3cas8ecse2cas7cas5cas6ecas1cas2* was present in two strains of *V. gazogenes*, CECT 5068 and DSM 21264. The associated type I-E arrays consisted of 15 total spacers (13 and 2 spacers, respectively), however no protospacer matches were identified. We identified a canonical type I-E system in *V. harveyi* ATCC 43516 with 37 spacers. In *S. sharmensis* DSM 18182, the type I-E systems had 79 spacers with protospacer targets in *Salinivibrio* phage SMHB1 (Additional file 2: Table S4).

### CRISPR-Cas type I-E system present within an excisable genomic island GI-24

**Fig. 8:**
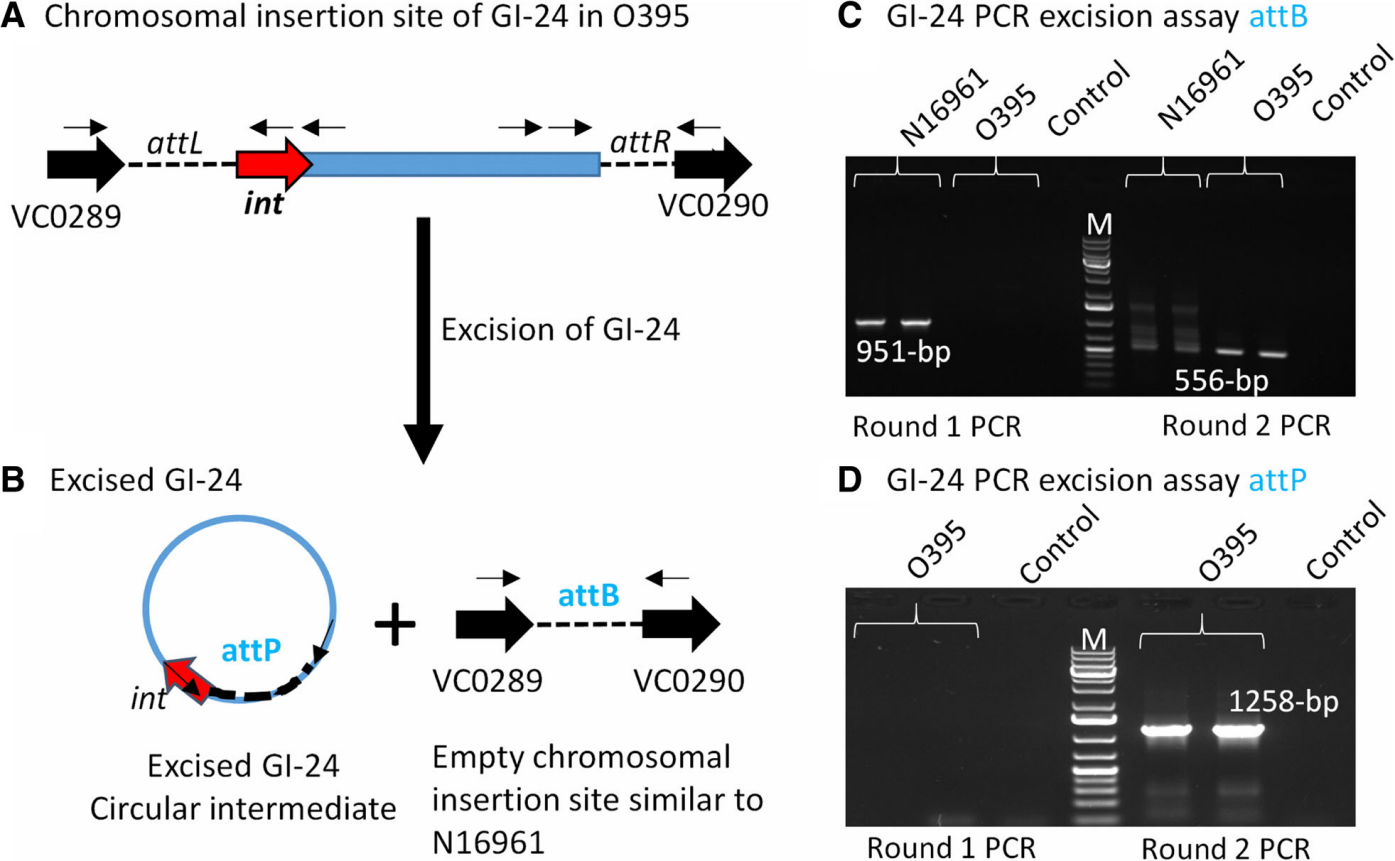
CRISPR-Cas type I-E island GI-24 excision assays. **a** Chromosomal insertion site of GI-24 showing att sites (broken lines), PCR primers (thin arrows), ORFs (block arrows), black ORFs indicate core ORFs, red ORF indicates integrase. **b** After excision of GI-24 from the chromosome a circular intermediate (CI) is predicted to form and an empty insertion site is present within the chromosome. **c** PCR products from an attB assay to detect excision of GI-24 from strain O395. In the second round of PCR, we used the first round of PCR cocktail as a template to detect a band. **d** PCR products from an attP assay to detect CI from O395 detecte after round 2 PCR

Based on our in silico analysis, 88% of the type I-E systems present in *Vibrionaceae* were carried on a MGE, including those present in GI-24, which was present in *V. cholerae*, *V. metoecus* and *V. albensis* (Fig. 7a). GI-24 contained an integrase required for site specific integration and conserved *attL* and *attR* sites that mark integration at the ends of GI-24 (Fig. 8a). The presence of integrase also suggests that the island can excise from the chromosome and be mobilized as a unit. To further investigate this, we performed a GI-24 excision assay that we have used previously to detect excision of several genomic islands in *V. cholerae* [36, 42, 43, 59]. A two-stage nested PCR approach was used to examine excision of GI-24 in *V. cholerae* classical strain O395 by detecting both a circular intermediate (attP) of the excised GI-24 and an empty GI-24 chromosomal insertion site (attB) after excision (Fig. 8b). We used *V. cholerae* N16961, which does not contain GI-24, as a control strain for the attB excision assay, which detects an empty insertion site in the chromosome. Using genomic DNA isolated from overnight cultures as template, we detected a PCR product of the expected size in an attB assay in N16961 in the first PCR reaction, but no product was detected for O395. This could suggest that in O395 excision does not occur or that it occurs at very low rates. Therefore, using as template the PCR reaction from round 1, we performed a second PCR attB reaction with a second primer pair. In this assay, we detected an attB PCR product from O395 indicating that GI-24 can excise but does so at low rates (Fig. 8c). To detect the GI-24 circular intermediate attP, we performed PCR using attP primers, after the first round of PCR, no product was produced but after the second round of PCR using the PCR cocktail from the first round as template, the expected attP PCR band was present for O395 demonstrating excision of GI-24 (Fig. 8d). These data show that the type I-E system is part of GI-24 and can be excised with the entire region, a likely first step in its transfer. Phylogenetic analysis was performed to determine whether the integrase and *cas8e* genes shared a common evolutionary history. There were a limited number of polymorphic sites among the strains examined, however in both trees *V. metoecus* formed a divergent branch from *V. cholerae* strains. Whereas eight *V. cholerae* strains shared identical clustering patterns in both trees (Additional file 1: Figure S6).

The type I-E system present in *V. harveyi* ATCC 43516 was carried on an 85-kb region inserted between LA59_08695 and LA59_08700, with respect to *V. harveyi* ATCC 33843, which lacked the region (Fig. 7d). The 85-kb region had a GC content of 40%, compared to 45% for the whole genome, however no integrase or transposase genes were identified. The region also contained genes for a type three secretion system (T3SS) (Fig. 7d). In *P. profundum* SS9 and *V. halioticoli* NBRC 102217, the type I-E system was identified within a homologous conjugative plasmid suggesting horizontal transfer between these distantly related species.

### Phylogenetic analysis of Cas8e proteins

The Cas8e protein sequences were aligned and a neighbor-joining tree was constructed. The branching patterns demonstrates the presence of six major clades designated I to VI (Additional file 1: Figure S7). We identified 12 *V. cholerae* biotype classical strains that contained highly homologous Cas8e proteins that clustered in lineage I with a Cas8e protein from *V. metoecus* strain YB5B06 and two Cas8e proteins from two *V. albensis* strains (Additional file 1: Figure S7). Divergent but related to this group were Cas8e proteins from two strains of *V. azureus* in lineage II. The next three divergent lineages, III, IV and V grouped Cas8e proteins based on the genus and species they were present in. Lineage III consisted of Cas8e proteins from 4 strains of *V. gazogenes* and one strain each of *V. spartinae and V. ruber* that were all highly related. Lineage IV was comprised of Cas8e from *V. parahaemolyticus* and *V. harveyi* clustered together and branching with these were Cas8e from two *Photobacterium* species (Additional file 1: Figure S7). Lineage V was comprised of Cas8e from 8 strains of *Salinivibrio* and one strain of *Photobacterium galatheae*. Finally, clade VI consisted of the two most divergent Cas8e proteins from *V. halioticoli* NBRC 102217 and *P. profundum* SS9, which contained a variant type I-E system carried on a plasmid.

### Type I-C CRISPR-Cas systems in *Vibrionaceae*

Previously, we identified a type I-C system in *V. metschnikovii* CIP 69.14 [36]. We used the Cas proteins from this species as seeds in BLAST searches to identify putative systems in the *Vibrionaceae*. This analysis identified type I-C CRISPR-Cas systems in 12 species; *V. metschnikovii, V. cidicii*, *V. hangzhousensis*, *V. navarrensis*, *P. aquimaris*, *P. marinum*, *V. anguillarum*, *V. salilacus*, *V. fujianensis*, *Vibrio sp*. V03-P4A6T147, *Salinivibrio sp.* DV, and *Photobacterium sp*. CECT 9192 (Additional file 2: Table S5). All type I-C systems identified, with the exception of the one present in *V. anguillarum*, contained the canonical CRISPR-Cas type I-C *cas* gene arrangement and a type I-C 32-bp direct repeat (Additional file 2: Table S5).

In *Vibrio* sp. V03-P4A6T147 and *V. hangzhouensis* CGMCC 1.7062, we were unable to identify CRISPR arrays due to short contig sequences. Across the remaining species there were a total of 491 spacers identified, and each had a conserved type I-C PAM. In *P. marinum*, there were two CRISPR arrays flanking the type I-C *cas* gene cluster each with a type I-C repeat. The CRISPR arrays ranged in size from 2 spacers up to 179 spacers present in *Salinivibrio* sp. DV, the largest array identified in this study (Additional file 2: Table S5). Protospacer targets were identified for 31 spacers from a total of 491 and of these 31 targets 16 were hits to the *Salinivibrio* phage SMHB1.

### Type I-C CRISPR-Cas system present within a Tn7-like transposon

**Fig. 9:**
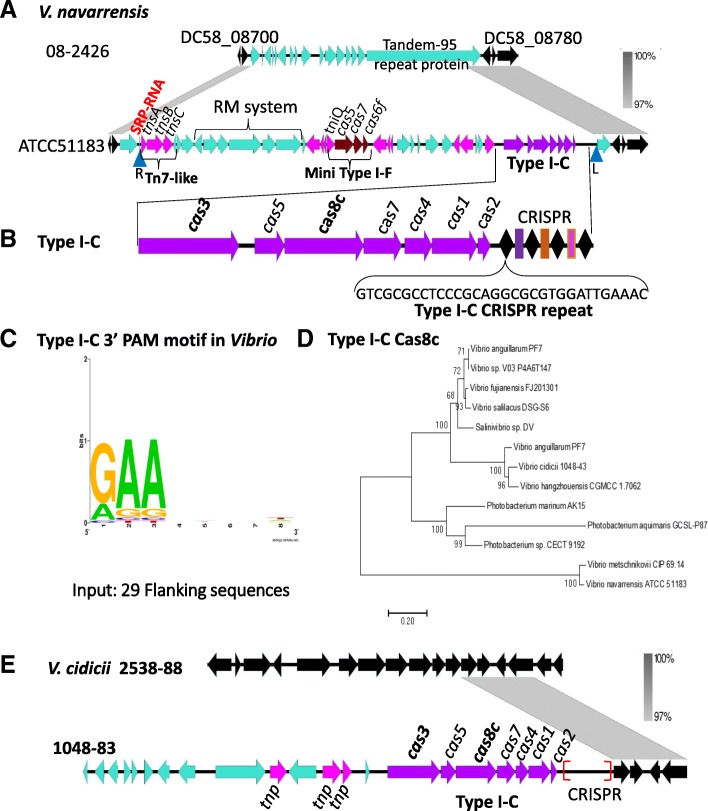
A Tn7-like transposon carrying a type I-C CRISPR-Cas system in *V. navarrensis.* **a** The type I-C CRISPR-Cas system is present within a mini type I-F-carrying Tn7-like transposon in *V. navarrensis* ATCC 51183 that is absent in *V. navarrensis* 08–2426. **b** Type I-C *cas* genes and CRISPR repeat in *V. navarrensis* ATCC 51183. **c** PAM identified using protospacer sequences. **d** A neighbor-joining tree of Cas8c from all of the type I-C systems identified. **e** Type I-C system in *V. cidicii* 1048–83 was inserted at ATY37_RS07420 relative to strain 2538–88 that lacked the region. Gene comparisons were constructed in Easyfig and gray scale bars indicates nucleotide homology. The core genes are colored in black, Tn7-like transposon and other transposase genes are in pink. Black diamonds in the CRISPR represent direct repeats and colored boxes represent spacers. Blue inverted triangles represent right end and left end attTn7 insertion sites

The type I-C system in *V. navarrensis* ATCC 51183 was present within a 53-kb region that was inserted within a Tandem-95 repeat protein, with respect to *V. navarrensis* 08–2426, which lacked the region (Fig. 9a). This 53-kb region in ATC51183 contained at least 11 different transposase genes, which flanked three different modules within the region; a restriction modification (RM) system, a mini type I-F system *tniQcas5cas7cas6* and a complete type I-C system with a CRISPR array. A Tn7-like transposon (*tnsABC*) was present at the 5′ end of the island at a SRP-RNA insertion site as described in *V. cholerae* and *V. parahaemolyticus*. We identified attTn7 sites that encompassed a RM system, the mini type I-F system and the complete CRISPR-Cas type I-C system (Table 1). A region within the element also contained two copies of a reverse transcriptase (RT) with group II intron origin (RT-G2_intron), a P-loop NTPase (TniB), and a protein with a TIR-like domain, which were also flanked by transposase genes. The type I-C CRISPR-Cas system contained a CRISPR array with a type I-C repeat and the PAM motif was also identified (Fig. 9b and c). Of the four sequenced *V. navarrensis* genomes only ATCC51183 contained this region.

In *V. cidicii* 1048–83, the type I-C system is present within a 25-kb region that contains three transposases genes and had a GC content of 40%, compared to 48% GC content for the entire genome (Fig. 9e). In *V. anguillarum* PF7, *V. hangzhouensis* and *P. aquimaris,* the type I-C systems was within a region that contained both transposases and integrase genes. However, several strains of *Vibrio, Photobacterium* and *Salinivibrio* contained only a complete type I-C system integrated within the genomes with no additional genes present suggesting it was the sole acquisition at the insertion site (Additional file 1: Figure S8). Overall it appears that the CRISPR-Cas systems in these species were acquired as distinct unit or modules and not within any identifiable MGE.

Phylogenetic analysis of the Cas8c proteins present in *Vibrionaceae* showed that *V. metschnikovii* and *V. navarrensis* Cas8c proteins were closely related to each other but were the most divergent Cas8c proteins and formed a separate highly divergent branch. Cas8c (Fig. 9d). In *V. metschnikovii* CIP69.14, the type I-C system was not associated with any MGE or signature MGE genes. In *V. anguillarum* PF7, two divergently transcribed *cas* gene clusters are present, *cas3cas5cas8cas7* and *cas3cas5cas8cas7cas4cas1cas2*. The Cas8c proteins from this species clustered within two distinct lineages, one with Cas8c from *V. salilacus, Vibrio* sp., *V. fujianensis* and *Salinivibrio sp*. DV and the second with Cas8c proteins from *V. cidicii* and *V. hangzhouensis*. The Cas8c proteins from three *Photobacterium* species clustered together with long-branch lengths indicating they are not closely related to each other. (Fig. 9d).

### Type II-B CRISPR-Cas system in *Vibrionaceae*

**Fig. 10:**
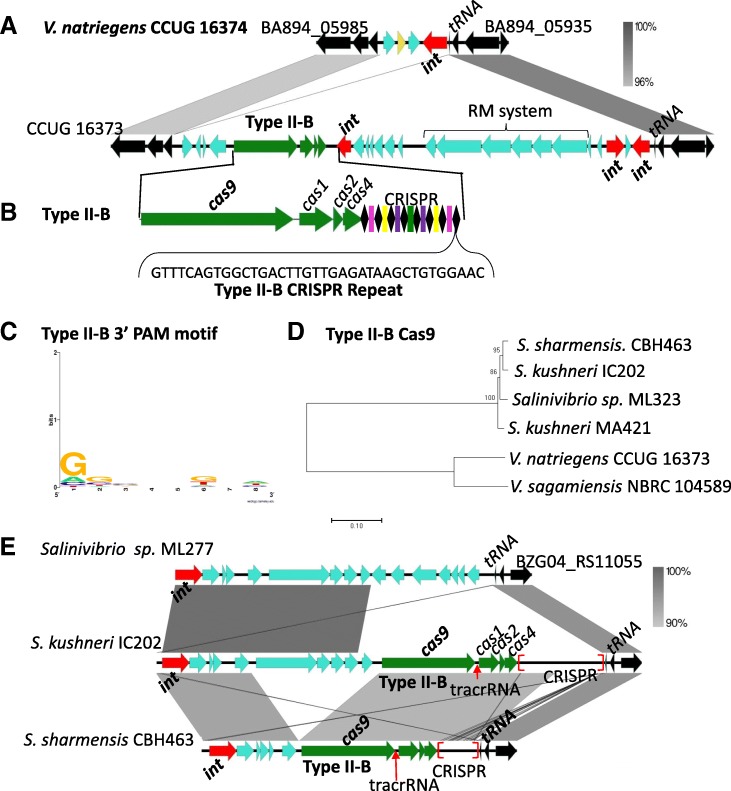
CRISPR-Cas type II-B systems in *Vibrionaceae*. **a** CRISPR-Cas type II-B system and a RM system present within an island in *V. natriegens* CCUG 16373 that is absent from strain CCUG 16374. **b** The type II-B system *cas* gene arrangement and a type II-B direct repeat sequences. **c** PAM for type II-B system. **d** A neighbor-joining phylogenetic tree of Cas9. **e** The CRISPR-Cas II-B system in *Salinivibrio kushneri* IC202 and *Salinivibrio sharmensis* CBH463 within an island at a tRNA locus relative to *Salinivibrio sp*. ML277. Genome comparisons were constructed in Easyfig and gray scale bars represent nucleotide homology. Black diamonds signify direct repeats and colored rectangles are spacers. The core genes are colored in black, transposases are colored in pink and integrases in red. Red brackets indicate the genomic location of CRISPR

Currently, no type II CRISPR systems have been characterized in *Vibrionaceae*. A recent study showed *Legionella pneumophila* contained a type II-B CRISPR-Cas systems, therefore we used this Cas9 as a seed to examine *Vibrionaceae* [60]. Five species were identified that contained a homolog of Cas9: *V. natriegens* CCUG 116373, *V. sagamiensis* NBRC 104589, *S. sharmensis* CBH463, two strains of *S. kushneri*, and *Salinivibrio sp.* ML323. All six strains were found to have the complete type II-B *cas* gene cluster of ***cas9****cas1cas2cas4* (Additional file 2: Table S6; Fig. 10).

Analyzing the CRISPR array, a type II-B system repeat sequence of 37-bp was identified in *Vibrio* and in *Salinivibrio* strains (Fig. 10b). We used CRISPRone to detect the trans-activating crRNA (tracrRNA), which is usually located between the *cas* genes and CRISPR array region and is complementary to the repeat sequence of the type II-B system, allowing it to pair with the repeat fragment of the pre-crRNA for interference [61]. We identified the tracrRNA downstream of the *cas1* in three out of the six strains analyzed as shown for *S. kushneri* IC202 and *S. sharmensis* CBH463 (Fig. 10e). The inability to detect the tracrRNA in the other three strains could be due to the threshold of 15 nucleotide match and at most two mismatches for the paring length set by the program [62]. Spacer analysis identified from 3 to 51 spacers among the strains with a total of 130 spacers and 15 putative protospacers were identified (Additional file 2: Table S6). Using these protospacers, we were able to identify the PAM sequence for these II-B systems and found it to be a 3’NGG 5′ (Fig. 10c), which is in agreement with what was previously shown in *Francisella novicida* [63].

Phylogenetic analysis based on the Cas9 obtained from the 6 strains demonstrated two major clades. Clade separation was genus specific: Clade I contains species belonging to *Salinivibrio* and showed highly related Cas9 proteins among three species. Divergent from these were Cas9 proteins in clade II from two *Vibrio* species (Fig. 10d).

### CRISPR-Cas type II-B systems present within MGEs

The type II-B system in *V. natriegens* CCUG 16373 was present within a 30-kb region that was inserted adjacent to a tRNA-Met locus that was absent from *V. natriegens* CCUG16374. (Fig. 10a). The 30-kb region contained a restriction modification system and three integrases, one of which was adjacent to the tRNA locus suggesting site specific integration (Fig. 10a). Within two *Salinivibrio* species, the type II-B system is also present within a genomic island that contains an integrase and is inserted at a tRNA locus (Fig. 10e).

### Type III CRISPR-Cas systems in *Vibrionaceae*

We used the Cas10 protein from the putative hybrid type III-B/I-F system to determine whether other species contained type III systems within *Vibrionaceae*. We identified 15 species that contained a type III system (Additional file 2: Table S7). Based on *cas* gene arrangement and *cas* gene homology, three subtypes were identified: type III-A, type III-B, and type III-D (Additional file 2: Table S7). In addition to these subtypes, we also uncovered a hybrid type III-B/I-F hybrid system in *V. palustris* CECT 9027 and *Salinivibrio* sp. DV (Additional file 2: Table S7). Interestingly the type I-F direct repeat in *Salinivibrio* sp. DV was identical to the repeat present in *V. metoecus* YB4D01 and *V. cholerae* (Additional file 2: Table S7). This suggests a common origin in distantly related species and recent horizontal transfer between these genera. In addition, we identified a type III-B system in *V. spartinae* CECT 9026 with three type I-F CRISPR arrays (Additional file 2: Table S7). In *Salinivibrio sp*. MA351, we identified a III-B system followed by a type I-F array but this system also clustered with a complete type I-F system (Additional file 2: Table S7).

The genome sequence for four *V. gazogenes* strains ATCC 43941, ATCC 43942, CECT 5068 and DSM 21264 each contained at least one type III system. *Vibrio gazogenes* ATCC 43941 and ATCC 43942 harbored identical type III-B systems on chromosome 1 with *cas2cas1* divergently transcribed from *hphpcmr1****cas10****cmr3cmr4cmr5cmr6* with two CRISPR arrays, one at each end of the *cas* gene clusters (Additional file 1: Figure S9A). Strains CECT 5068 and DSM 21264 harbored a homologous type III-B system with two CRISPR arrays and is found on chromosome 2 (Additional file 1: Figure S9B). These strains also contained a type III-A system with two arrays, one at each end of the *cas* loci (Additional files 2 and 1: Table S7; Figure S9C).

We identified an additional five strains with a type III-A system containing the *cas* gene arrangement of *cas10cas7cas5cas7cas1cas2* (Additional file 2: Table S7). The type III-A system in these strains contained one type III-A CRISPR array, with the exception of *P. aphoticum* JCM 19237, which contained two type III-A CRISPR arrays. Seven strains containing a type III-D system were also identified containing *cas10csm3csx10csm3csx19cas7cas6* along with *cas1cas2* genes in close proximity (Additional file 2: Table S7).

In 18 of the strains with a type III system characterized in this study, *cas1cas2* were present. Of note was the presence of a reverse transcriptase (RT) domain in 14 of the 18 Cas1 proteins identified. In type III-A system of *V. gazogenes* CECT 5068 and DSM 212464, the Cas1 protein is fused with RT and Cas6 domains. In 11 strains with either a type III-A, III-B or III-D system, only RT and Cas1 domains are fused. In *P. aphoticum* JCM 19237, the RT encoding gene is adjacent to the *cas1*. These RT containing Cas1 proteins have been shown previously to be primarily found in proximity to type III systems, are not specific to any subtype, and function autonomously [64]. In addition, the reverse transcriptase activity of the RT-Cas1 domain is required for spacer acquisition from RNA [65].

Neighbor-joining trees were constructed from the Cas1 domain sequences and the Cas10 proteins to determine the evolutionary history of these proteins. In clade I of the Cas1 domain tree, the seven strains containing a type III-A system are clustered (Additional file 1: Figure S10A). This clade contained two *V. gazogenes* strains with a *cas1* gene with *cas6* and retron domains and are distantly related to *cas1* genes from *Photobacterium* and *Vibrio* species. In clade II, the Cas1 from the four strains containing a type III-B cluster together from 4 *V. gazogenes* strains (Additional file 1: Figure S10A). In these strains, the Cas1 is directly next to but transcribed divergently from the type III-B system *cas* genes and contains a retron domain. Clade III consists of three *Vibrio* species with a type III-D system. The Cas1 from these three strains has a fused RT domain (Additional file 1: Figure S10A). Clade IV contains four species with a type III-D system that formed the most divergent cluster with a Cas1 only domain. This clade is highly divergent, characterized by long-branch lengths (Additional file 1: Figure S10A).

In the Cas10 tree from the strains containing Cas1, the proteins are separated based on the subtypes, with all type III-A clustered together, type III-B clustered together and all type III-D clustered together (Additional file 1: Figure S10A-B). The Cas10 from the seven strains containing a type III-D system are much more closely related and cluster in one single clade (Additional file 1: Figure S10B). In the Cas10 tree, proteins from the type III-B are the most divergent (Additional file 1: Figure S10A-B). These data suggest that each type III systems share a similar evolutionary history which is not the case within the Cas1 domain tree.

### CRISPR-Cas type III systems within MIGEs

**Fig. 11:**
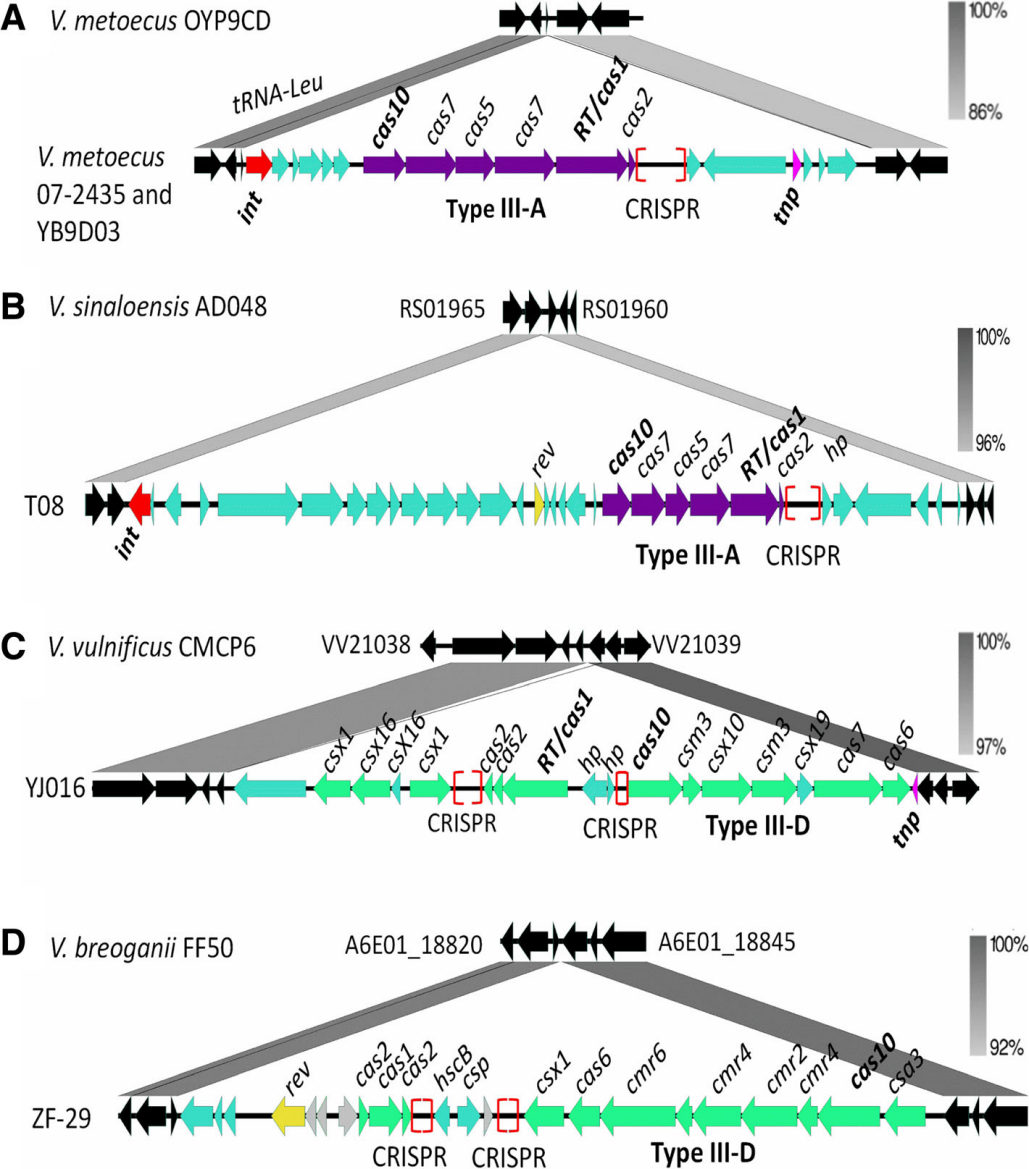
CRISPR-Cas type III systems within MIGEs. **a** *V. metoecus* strains 07–2435 and YB9D03 contain a type III-A CRISPR-Cas system carried on an island inserted at a tRNA-Leu locus in OYP9CD, which lacks the island. **b** *V. sinaloensis* T08 contains a type III-A system on an island inserted between A6E01_118830 and A6E01_18840 relative to strain AD048. **c** *V. vulnificus* YJ016 contains a type III-D CRISPR-Cas system identified on an island inserted between VV21038 and VV21039 relative to *V. vulnificus* CMCP6. **d** *V. breoganii* ZF-29 contains a type III-D CRISPR-Cas system on an island which also contains a putative resolvase. The island is inserted between A6E01_118830 and A6E01_18840 of strain FF50. Genomic comparisons were constructed using Easyfig with gray scale bars representing nucleotide homology. The core genes are colored in black, transposases are colored in pink and integrases in red. Red brackets indicate the genomic location of CRISPR

As described above, the CRISPR-Cas type III-B/I-F putative hybrid system was associated with a prophage in both *V. cholerae* and *V. metoecus***.** In *V. metoecus* 07–2435, a type III-A system was carried on a 26-kb island, which contains an integrase at the 3′ end of the island and a transposase towards the 5′ end of the island (Fig. 11a). The island was inserted at a tRNA-Leu locus and the region has a GC content of 44%, compared to 47% for the genome. In *V. sinaloensis* T08 a type III-A system was present on a 46-kb island flanked at the 3′ by an integrase and is inserted at an L-threonine 3-dehydrogenase with respect to strain AD048 which lacked the island (Fig. 11b). The region had a GC content of 43% compared to 46% across the entire genome. In *V. vulnificus* YJ016, a type III-D is carried on a 22-kb island and is inserted in between VV2_1039 and VV2_1038 with respect to *V. vulnificus* CMCP6 that lacked the region (Fig. 11c). We also identified a transposase associated with this island. Finally, in *V. breoganii* ZF-29, the III-D system is present on a 22-kb island between A6E01_18830 and A6E01_18835 relative to strain FF50, which lacks the entire region (Fig. 11d). The data suggests that these CRISPR-Cas systems are present within regions recently acquired but does not indicate that they were acquired with the element or were added later.

Phylogenetic analysis of all the Cas10 proteins identified demonstrated that these systems are highly divergent from one another. One exception is the Cas10 from the hybrid type III-B/I-F systems associated with a prophage, which all clustered together (Additional file 1: Figure S11). Branching from these type III-B/I-F hybrid systems were *V. palustris* CECT 9027, *V. spartinae* CECT 9026 and *Salinivibrio sp.* MA351 which all have a type III-B system and I-F arrays. In clade II, Cas10 proteins from *V. gazogenes* strains cluster together demonstrating homologous type III-B systems. Clade III is comprised of the seven strains containing a type III-A system, which are homologous to each other and encompasses *Photobacterium* and *Vibrio* species. Clade IV contains the diverse type III-D systems and comprise of Cas10 proteins from *Vibrio*, *Salinivibrio* and *Photobacterium* species (Additional file 1: Figure S11).

### Type IV CRISPR-Cas systems in *Vibrionaceae*

**Fig. 12:**
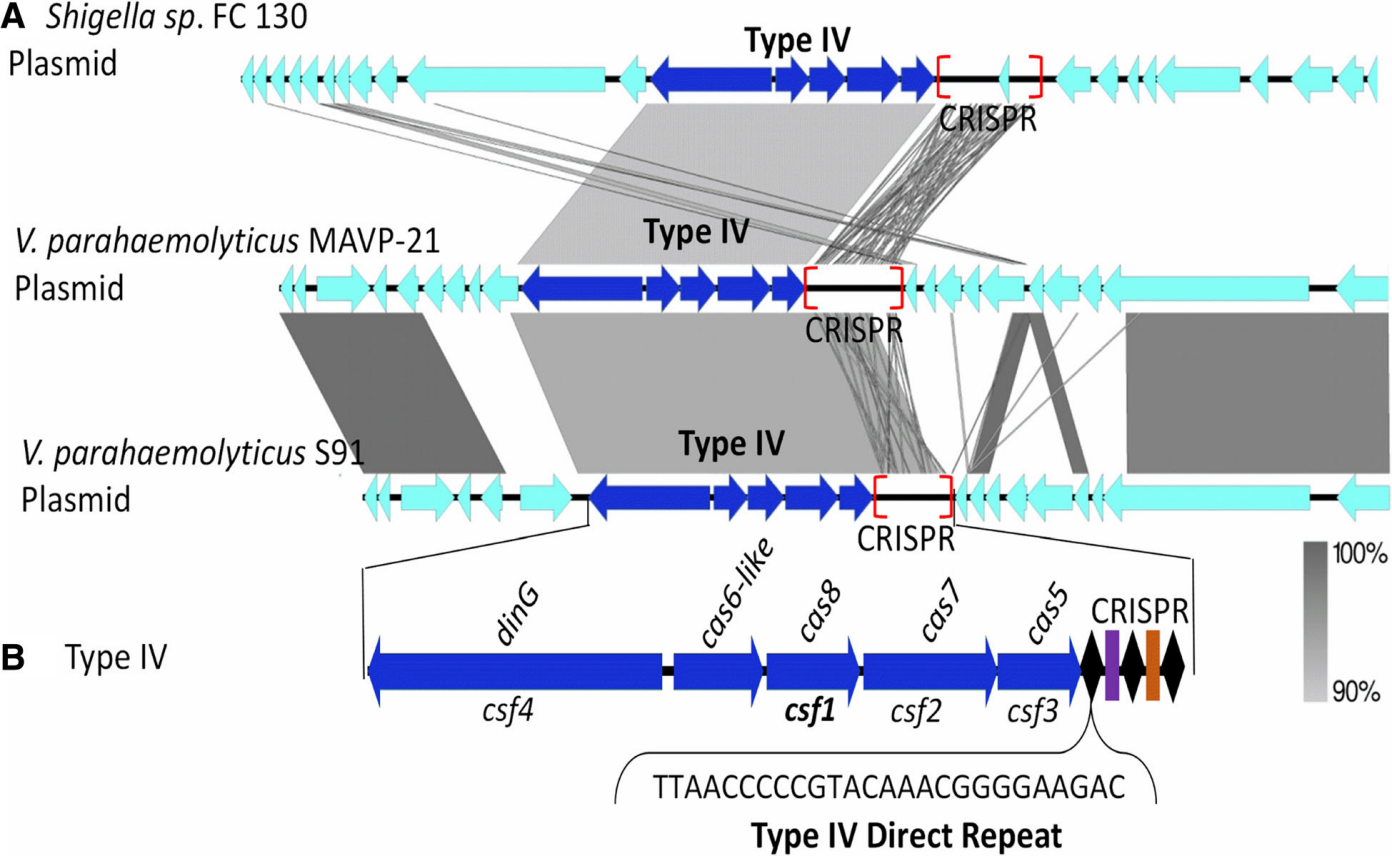
CRISPR-Cas type IV system on a plasmid. **a** Plasmid of *V. parahaemolyticus* MAVP-21 and *V. parahaemolyticus* S91 harbored a Type IV CRISPR-Cas system homologous to the one found in the plasmid of *Shigella sp*. FC 130. **b** The type IV systems have canonical type IV gene arrangement and the CRISPR contains canonical type IV direct repeat. Genomic comparisons were constructed using Easyfig with gray scale bars representing nucleotide homology. Black diamonds indicate direct repeats and colored rectangles indicate spacers

Type IV systems are rare and all have been discovered to be present on plasmids [15, 26]. In our analysis of *V. parahaemolyticus*, we identified two strains containing type IV systems also carried on plasmids (Fig. 12a). These systems were homologous to the type IV system on a plasmid in *Shigella* sp. FC 130 and consisted of *csf4cas6-likecsf1csf2csf3* gene arrangement (Fig. 12b). In *V. parahaemolyticus* MAVP-21, the type IV system has an associated CRISPR array consisting of 24 spacers and a direct repeat sequence of 5’ACTCTTTAACCCCCTTAGGTACGGG 3′. A sequenced plasmid, S91, also carried a type IV system associated with an array with 19 spacers and a direct repeat sequence of 5’ TTAACCCCCGTACAAACGGGGAAGAC 3′. Between the two CRISPR arrays, we did not identify spacer targets.

## Discussion

CRISPR-Cas systems are an adaptive, bacterial defense system against invading DNA (and RNA in some cases) such as phages and plasmids. Most of the studies of these systems have been conducted in *Escherichia coli, Pseudomonas aeruginosa* and several species of *Streptococcus,* leaving many families of bacteria unexamined [12, 29, 66, 67]. In this study, comparative genomics and bioinformatics analyses were used to identify the types of CRISPR-Cas systems present in marine species belonging to the family *Vibrionaceae*. In multiple species, different types of CRISPR-Cas systems were identified, including novel variants. The variation was marked by the presence of different *cas* genes and different gene arrangements.

In *V. cholerae*, canonical and variants of type I-F were identified within genomic islands and mini type I-F systems were present within Tn7-like transposons. The mini type I-F system was also identified in a large number of *V. parahaemolyticus* strains as well as many additional species. A previous study has suggested that the Tn7-like transposons have coopted the mini type I-F CRISPR-Cas systems to identify and target its insertion site [44]. The Tn7-like transposon lacks key genes required for its function, specifically TnsE and TnsD homologues required for integration, and the mini CRISPR-Cas system lacks genes required for target cleavage (*cas3*) and spacer acquisition (*cas1* and *cas2*). Peters and co-workers hypothesize that the CRISPR containing transposon function together to form a functional element that allows for target integration into MGE and/or movement within a genome [44]. In *V. parahaemolyticus*, a significant pathogen of humans, the main virulence mechanism, a T3SS-2 system, is present within a CRISPR-Cas-carrying Tn7-like element. T3SS-2 systems are large genomic regions of > 80-kb and are only present in human pathogenic strains and are absent from environmental isolates [49, 52, 53]. T3SS-2 systems have been identified at different genome locations depending on the strain analyzed and the mechanism of acquisition and insertion is unknown. Our analysis shows that the T3SS-2 systems in *V. parahaemolyticus* are all part of CRISPR-carrying Tn7-like transposons and indicate a mechanism by which these virulence factors can be mobilized, which needs to be investigated further.

A putative hybrid system consisting of type III-B genes and *cas6f* followed by a type I-F array, was present in eight *V. cholerae* strains and two *V. metoecus* strains. The strains were isolated between 1974 and 2012 from three continents and each strain contained a unique array with between 12 and 50 spacers. Although further experimental evidence is required, the type III-B/I-F could be a true hybrid system. It has been proposed that extensive recombination events within the CRISPR-Cas region may lead to the formation of a hybrid system [16, 68]. The type III-B/I-F hybrid system is associated with a prophage region and thus may explain the high similarity between the systems found in a few strains in two species. Although one cannot rule out the possibility that the CRISPR-Cas systems is only co-localized with the prophage.

This study is the first, large scale genome analysis to characterize the CRISPR-Cas systems in the family *Vibrionaceae*, members of which are marine inhabitants that encounter huge amounts of viruses and thus would be good candidates for the presence of diverse CRISPR-Cas systems. In addition to type I-F systems, our analysis uncovered type I-E, I-C, III-A, III-B, III-D systems and system variants, as well as type II-B and type IV systems. Thus, there is a diversity of systems present among the different species, but their occurrence is infrequent suggesting that these systems are not a major contributor to bacterial fitness and survival. The multiple variations in the *cas* gene arrangements and content identified here suggests that gene rearrangements occur frequently and the systems are constantly in flux. In addition, our analysis demonstrates that the majority of systems identified in *Vibrionaceae* are associated with MGEs and that many of these elements carry additional cell defense systems mainly restriction modification systems and toxin-antitoxin systems. In these MGEs, CRISPR-Cas may be playing more of a role in protecting the MGE they are carried on rather than the strain they are present in, similar to the role of the type I-F system present in phages isolated from cholera stool samples [33, 37]. Phylogenetic analysis using marker Cas proteins suggests that within some species acquisition occurred multiple times, resulting in strains with multiple CRISPR-Cas systems of different system types, which leads to the question of whether some strains are more susceptible for uptake of these systems than others. Overall, the data shown that CRISPR-Cas systems were sporadic in distribution, and are not ancestral to any species of *Vibrionaceae*. In addition, the presence of these systems on MGEs carrying additional cargo genes such as virulence genes could suggest additional roles within the cell.

## Conclusions

The CRISPR-Cas repertoire is an ever-expanding defense system in bacteria. In *Vibrionaceae*, these systems are highly diverse with multiple sub-types and sporadic in distribution. The identification of protospacer targets suggests that these systems were active within this family. Importantly, the association of these systems with genomic islands, plasmids, phages, and transposons suggest a vector for their transfer amongst species. Furthermore, the mobility of these systems can lead to novel variant subtypes, which we have identified several examples of, including a type III-B/I-F hybrid system. This work opens the door for future studies in determining how CRISPR-Cas systems affect the survival of these bacteria and potentially novel functions of these systems.

## Methods

### CRISPR-Cas sequence analysis and predictions

The NCBI non-redundant protein sequences database was queried using BLASTp, initially using the Cas protein sequences previously identified in *V. cholerae* as well as signature Cas proteins identified in other species as seeds. A complete list of the bacterial strains within *Vibrionaceae* that contained a CRISPR-Cas system with a CRISPR array (repeat and spacer) and the accession number of the sequence contigs are shown in (Additional file 2: Table S8). The FASTA sequences containing putative CRISPR-Cas systems were downloaded from the NCBI genome database and searched for CRISPR arrays (repeats and spacers) using the CRISPRDetect software tool [69] and the CRISPRFinder tool [70]. The direct repeat sequences of putative CRISPR arrays determined by CRISPRDetect and CRISPRFinder were used as an input for CRISPRMap [71] to assign a type and subtype to each of the newly identified CRISPR arrays.

### Identification of putative protospacers

All CRISPR spacers identified by CRISPRFinder and CRISPRDetect were used as query for the CRISPRTarget program, using default parameters to identify the complementary protospacer sequence of each spacer [72]. A cutoff score of 22 was used for the analysis. Using Weblogo [73], we aligned the 3′ flanking sequences of the protospacer hits, comprised of 8-bp, to visualize the motif. Phage genomes were downloaded from the NCBI database and the distribution of protospacer hits were mapped onto the phage genome. Targeted gene loci were determined by examination of the target phage genome.

### Identification of MGEs

The genomic region surrounding the CRISPR-Cas systems were analyzed for the presence of markers of MGEs such genomic islands, phages, and transposons markers included integrases (*int*), transposases (*tnp*) and site specific attachment (att) sites. In addition the %GC content of each region was determined and compared to the overall %GC content of the chromosome. To identify regions of difference between strain with and without a CRISPR-Cas system, the sequence/contig with the CRISPR-Cas system was compared with related strains or species lacking the system, and figures were generated using Easyfig [74]. Phaster, a phage identification tool, was used to identify phage regions associated with CRISPR-Cas systems [54]. Minimal sized contigs where core genes were unable to be identified were excluded from MGE analysis.

### Comparative analysis of CRISPR-Cas systems

The Genbank sequences of CRISPR-Cas systems were downloaded from the NCBI genome database. The nucleotide sequence for these were compared using the Easyfig comparison tool. BLASTn, with default settings, was used for comparison, unless otherwise stated.

### Phylogenetic analysis of CRISPR-Cas systems

Evolutionary analysis was performed on Cas1, Cas6f, Cas8e, Cas8c, Cas9, and Cas10 proteins as well as integrase genes associated with CRISPR-Cas systems from species within the family *Vibrionaceae*. Additionally, evolutionary analysis was conducted using Cas1 and Cas10 sequences from the type III systems. Protein sequences were obtained from NCBI database and aligned using the ClustalW algorithm [55]. Aligned protein sequences were used to generate a Neighbor-Joining tree with a bootstrap value of 1000, and the Poisson model of substitution with pairwise deletion in MEGA7 [75].

### Genomic island GI-24 excision assays

PCR assays to detect both the empty site attB following excision of GI-24 as well as the circular intermediate attP of excised GI-24 were performed as previously described (Carpenter et al. 2015). DNA was isolated from *V. cholerae* O395 and N16961 from overnight liquid incubations using NucleoSpin Tissue kit (Macherey-Nagel, Duren, Germany) following the manufacturer’s instructions. In order to detect the excision, a nested two-stage PCR was performed to amplify the empty chromosomal site attB or the circular intermediate attP. The first round of PCR was performed in 20 μl reactions using 10 ng of isolated DNA as template using primer pairs: attB_R1L: agtttggtgcgggtatcaag and attB_R1R: gccactgcgtgactctgtta and for attP the following primers were used: attP_R1R: gctccctccttcaagtaccgctc and attP_R1L: gcgaaactgccaacgcacg. Following the first round of PCR, 1 μl of the reaction product was used as a template for a second round of PCR using attB primers_attBR2L aaagtgggcgagtagggtt and_attBR2R tctggacaccatcatgcaat and attP primers_attPR2R ccgatagcgacaatgacactgc and attPR2L gagacccttgcacccaatccatc. The PCR products were analyzed by gel electrophoresis on a 1% agarose gel stained with ethidium bromide. Experiments were performed with two biological replicates and three technical replicates.

## Additional files

Additional file 1: **Figure S1.** Distribution of CRISPR-Cas system types identified among *Vibrionaceae*. (A) Percentage break down of all the systems identified by type. Different colors are used to indicate different CRISPR-Cas systems. (B) Percentage of systems identified that are associated with mobile genetic elements. (C) Diversity of each system identified within the *Vibrionaceae*. **Figure S2.** Phylogenetic analysis of *cas1 and intV* from *V. cholerae* strains containing type I-F system. The *cas1* nucleotide sequences were examined for 21 type I-F systems characterized in *V. cholerae*. For the same 21 strains *intV* nucleotide sequences from VPI-6 were collected and analyzed. Aligned sequences were used to construct a neighbor-joining phylogenetic tree with bootstrap of 1000. **Figure S3.** Phylogenetic analysis of *cmr1*, *cas6f* and phage associated integrase. The nucleotide sequences for *cmr1* and *cas6f* from *V. cholerae* and *V. metoecus* strains containing the putative type III-B/I-F system were examined to construct a phylogenetic tree. Similarly, the nucleotide sequences of the integrase from the prophage associated with the putative type III-B/I-F system were used to construct a phylogenetic tree. Aligned sequences were used to construct a neighbor-joining phylogenetic tree with bootstrap of 1000. **Figure S4.** Protospacer targets from *V. cholerae* I-F systems to *Vibrio* phages. Protospacer targets identified within the I-F arrays of *Vibrio cholerae* were mapped to the genomes of Vibrio phages X29/phi2, phage Kappa, phage fs2, fs-1, and KSF1-phi. Genomes were constructed using EasyFig. **Figure S5.** CRISPR-Cas type I-F systems in *Vibrionaceae* are within MGEs. Comparative analysis of the genomic regions containing type I-F systems demonstrated their presence on genomic islands. (A) The CRISPR-Cas island in *V. metoecus* YB5B04 is absent from *V. metoecus* strain OYP8G12. (B) The type I-F system present within an island in *V. parahaemolyticus* A4EZ703 between VPA0712 and VPA0713 relative to RIMD2210633 that lacks the island. (C) CRISPR-Cas island in *V. vulnificus* 93 U204 is inserted relative to VV1_0636 and a tRNA in CMCP6 that lacks the island. (D) Comparative analysis of the genomic regions containing type I-F systems demonstrated their presence on genomic islands. Type I-F systems were identified in an island region in four *V. fluvialis* strains, which was absent in strain S1110. The genome comparisons and gene diagrams were drawn using EasyFig and the grayscale bars represent nucleotide homology. The core genes are colored in black, transposases are colored in pink and integrases are red. Red brackets indicate the genomic location of CRISPR. **Figure S6.** Phylogenetic analysis of *cas8e* and *int* from *V. cholerae* strains containing type I-E system within GI-24. The *cas8e* and associated *int* nucleotide sequences from *V. cholerae* were collected and analyzed using MEGA. Aligned sequences were used to construct a neighbor-joining phylogenetic tree with bootstrap of 1000. **Figure S7.** Phylogenetic analysis of type I-E Cas8e from *Vibrionaceae*. The Cas8e protein sequences were examined for all type I-E systems characterized in this study. Aligned sequences were used to construct a neighbor-joining tree with bootstrap of 1000. *Salinivibrio* are indicated by green squares and *Photobacterium* by light blue triangles. **Figure S8.** Genomic loci analysis of type I-C systems in *Vibrionaceae.* The genomic region containing type I-C systems identified in *Vibrionaceae* were analyzed. Gray scale bars represent nucleotide homology. The core genes are colored in black, transposases are colored in pink, resolvase in yellow and integrases in red. Red brackets indicate the genomic location of CRISPR. **Figure S9.** Type III systems found within *V. gazogenes*. (A) A type III-B system found in strains ATCC 43941 and ATCC 43942. (B) A type III-B system in strains DSM 21264 and CECT 5068. (C) The type III-A system present in DSM 21264 and CECT 5068 present on chromosome II. Genome comparisons were constructed in Easyfig and gray scale bars represent nucleotide homology. The core genes are colored in black, transposases are colored in pink and integrases in red. Red brackets indicate the genomic location of CRISPR. **Figure S10.** Phylogenetic analysis of Cas1 domain and Cas10. (A) Cas1 domain sequences and (B) Cas10 sequences from strains containing a type III system and a Cas1 protein were aligned in MEGA7 with clustalW to construct a neighbor-joining phylogenetic tree with bootstrap of 1000. **Figure S11.** Phylogenetic analysis of Cas10 from the family *Vibrionaceae*. Cas10 sequences were collected from 31 strains belonging to various species from the family *Vibrionaceae* containing the type III system. ClustalW was used to align the collected sequences and a neighbor-joining tree was built with bootstrap of 1000 using MEGA7. The protein sequences separated into four distinct clusters. The *Salinivibrio* are indicated by green squares and the *Photobacterium* are indicated by light blue triangles. (PPTX 2880 kb)

Additional file 2: **Table S1.** Type I-F systems in *V. cholerae*. **Table S2.** Type I-F systems in *Vibrionaceae*. **Table S3.** Type I-E systems in *V. cholerae*. **Table S4.** Type I-E systems in *Vibrionaceae*. **Table S5.** Type I-C systems in *Vibrionaceae*. **Table S6.** Type II-B systems in *Vibrionaceae.* **Table S7.** Type III CRISPR-Cas systems identified in *Vibrionaceae*. **Table S8.** CRISPR-Cas contig accession numbers for species in this study. (XLSX 55 kb)

## Abbreviations

att: Attachment sites
Cas: CRISPR associated proteins
CRISPR: Clustered Regularly Interspaced Short Palindromic Repeats
crRNA: CRISPR RNA
DRs: Direct Repeats, R end right end attachment site L end Left end attachment site
GI: Genomic Island
*int*: Integrase
MGEs: Mobile genetic elements
PAM: Protospacer adjacent motif
RM: Restriction modification
RT: Reverse transcriptase
T3SS: Type III secretion system
*tnp*: Transposase
tracrRNA: trans-activating crRNA
VPI: Vibrio pathogenicity island
