# Supplementary figures and images for: CRISPR-Cas systems are present predominantly on mobile genetic elements in Vibrio species
Source: BMC Genomics. 2019 Feb 4;20:105. doi: 10.1186/s12864-019-5439-1 (PMC6360697; doi:10.1186/s12864-019-5439-1)

## Slide 1
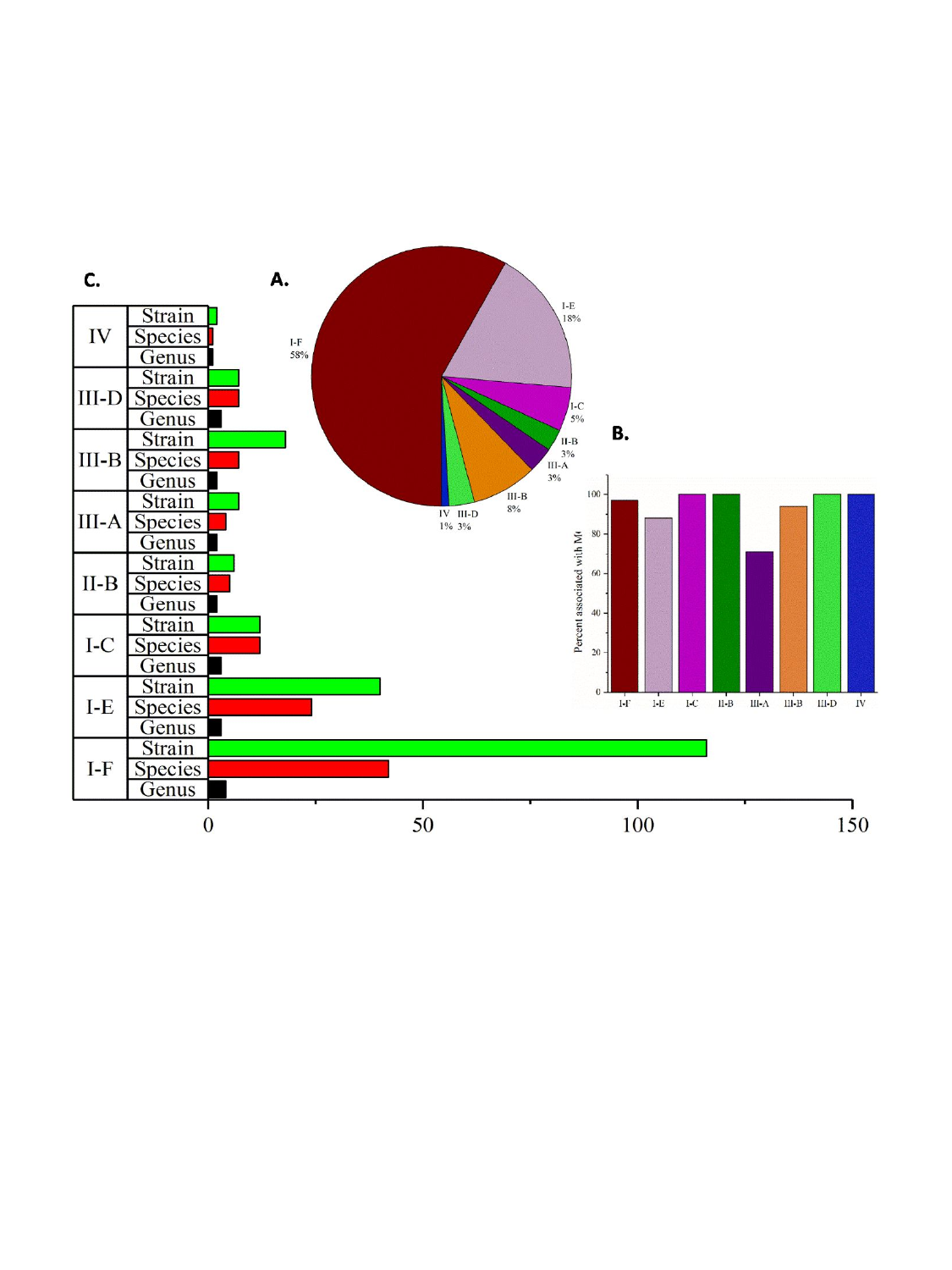

## Slide 2
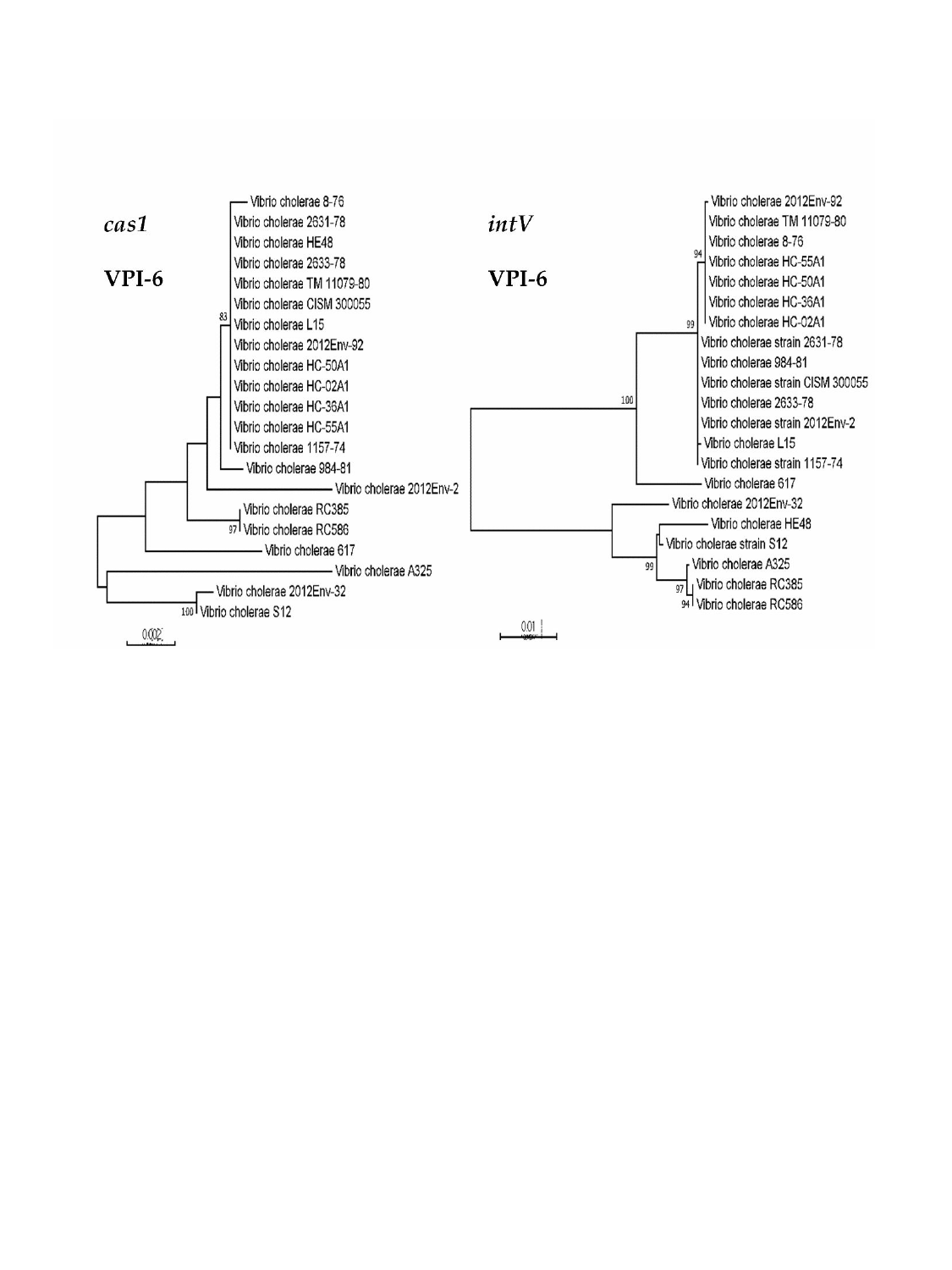

## Slide 3
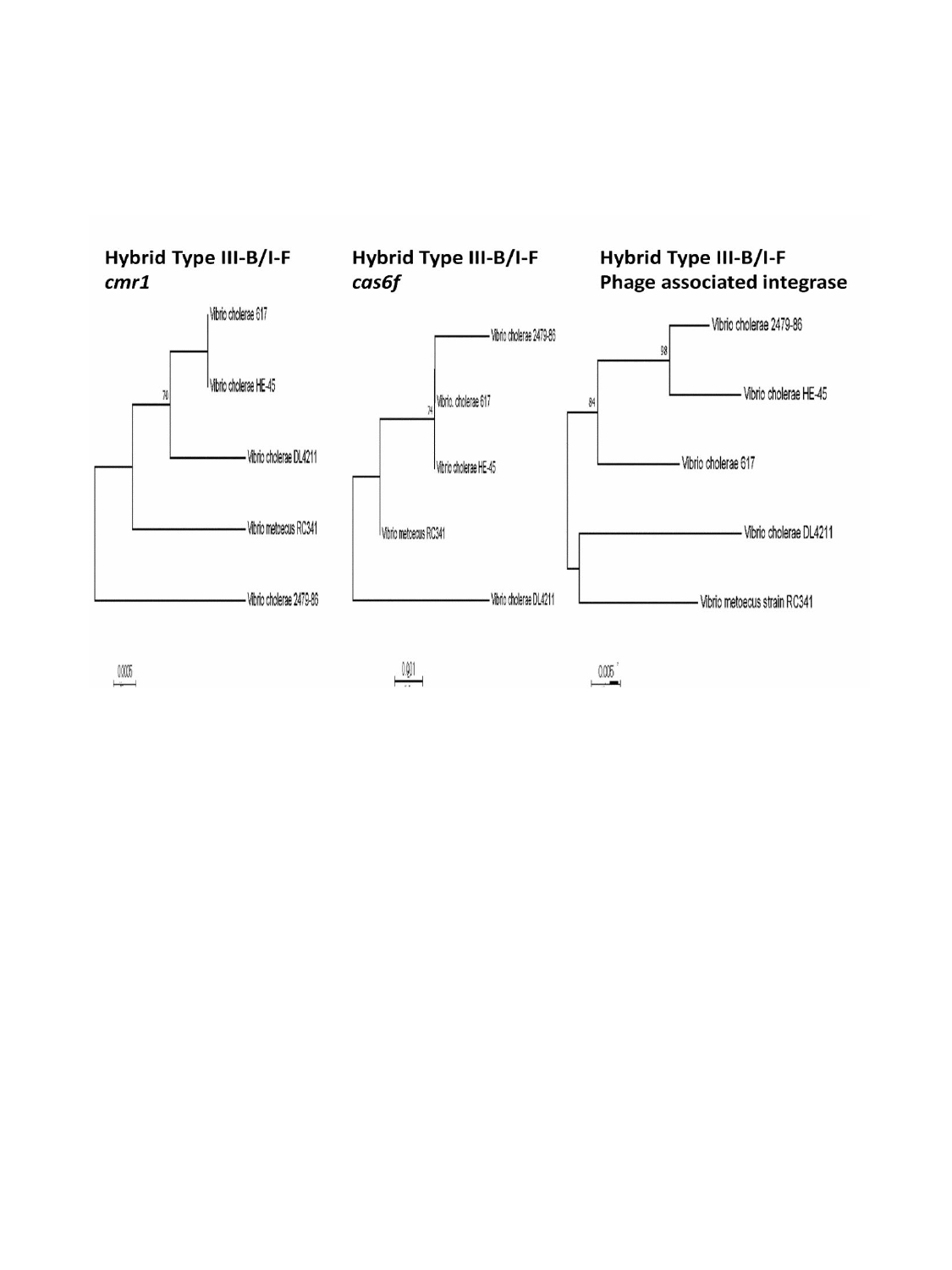

## Slide 4
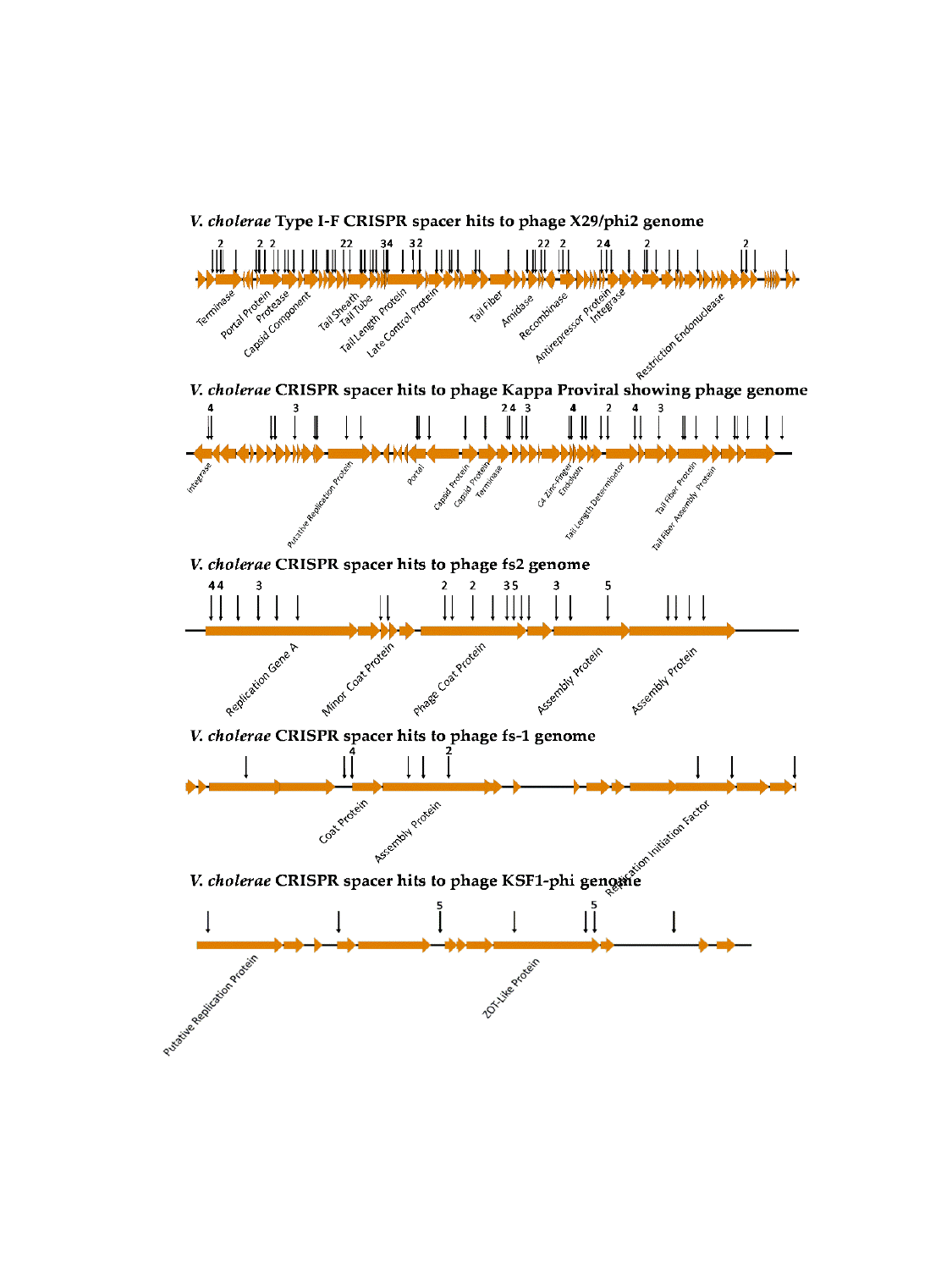

## Slide 5
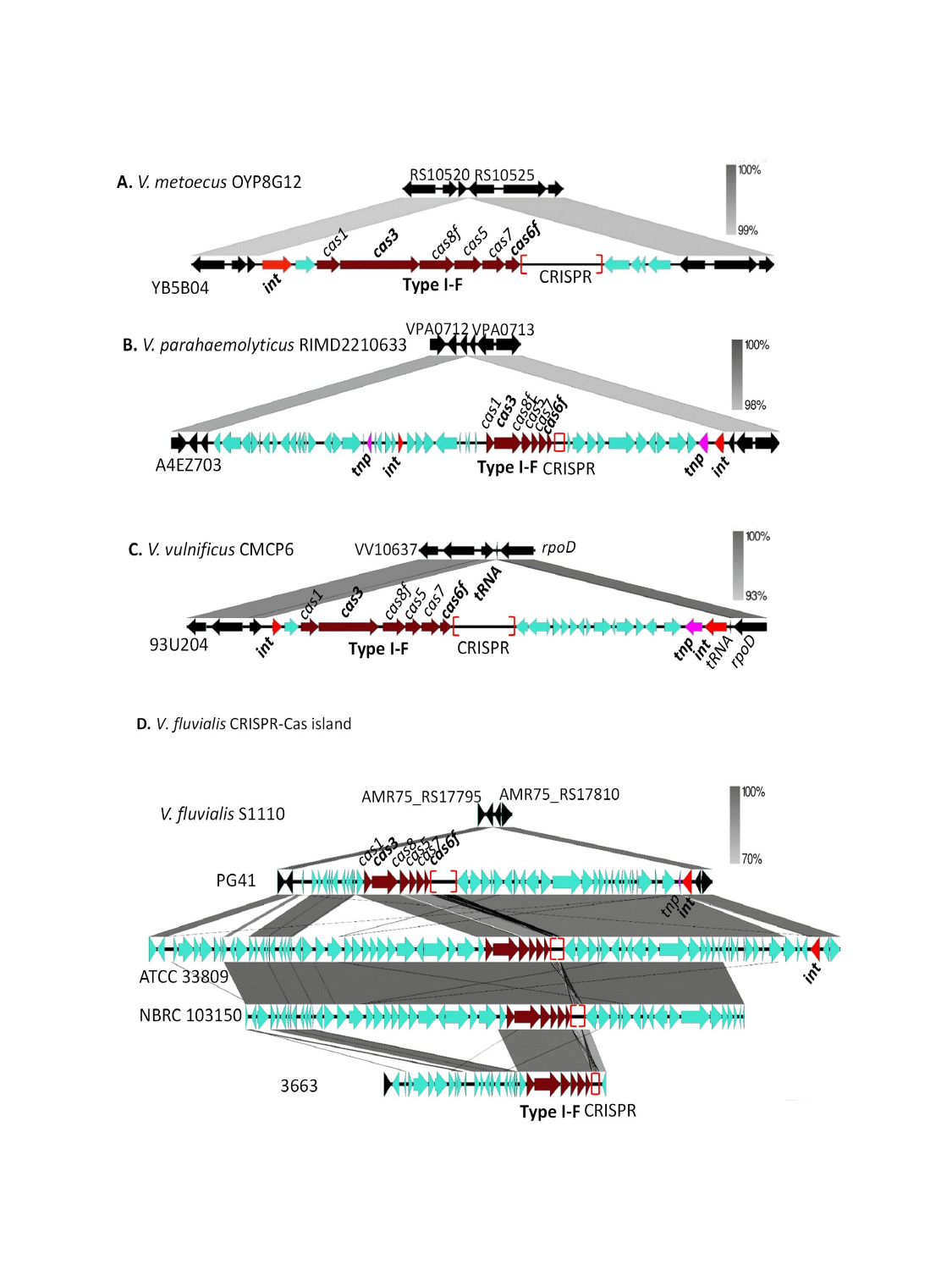

## Slide 6
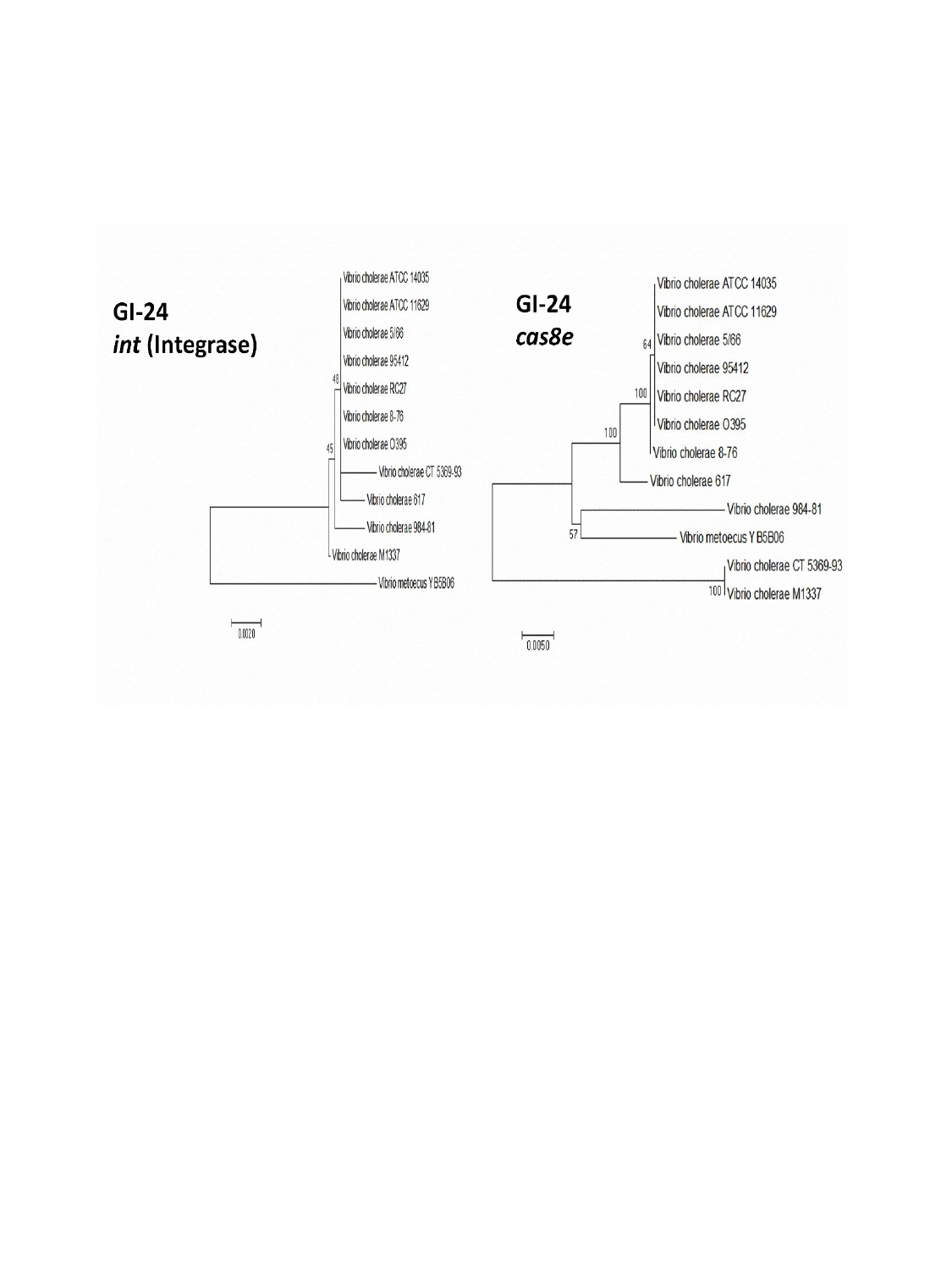

## Slide 7
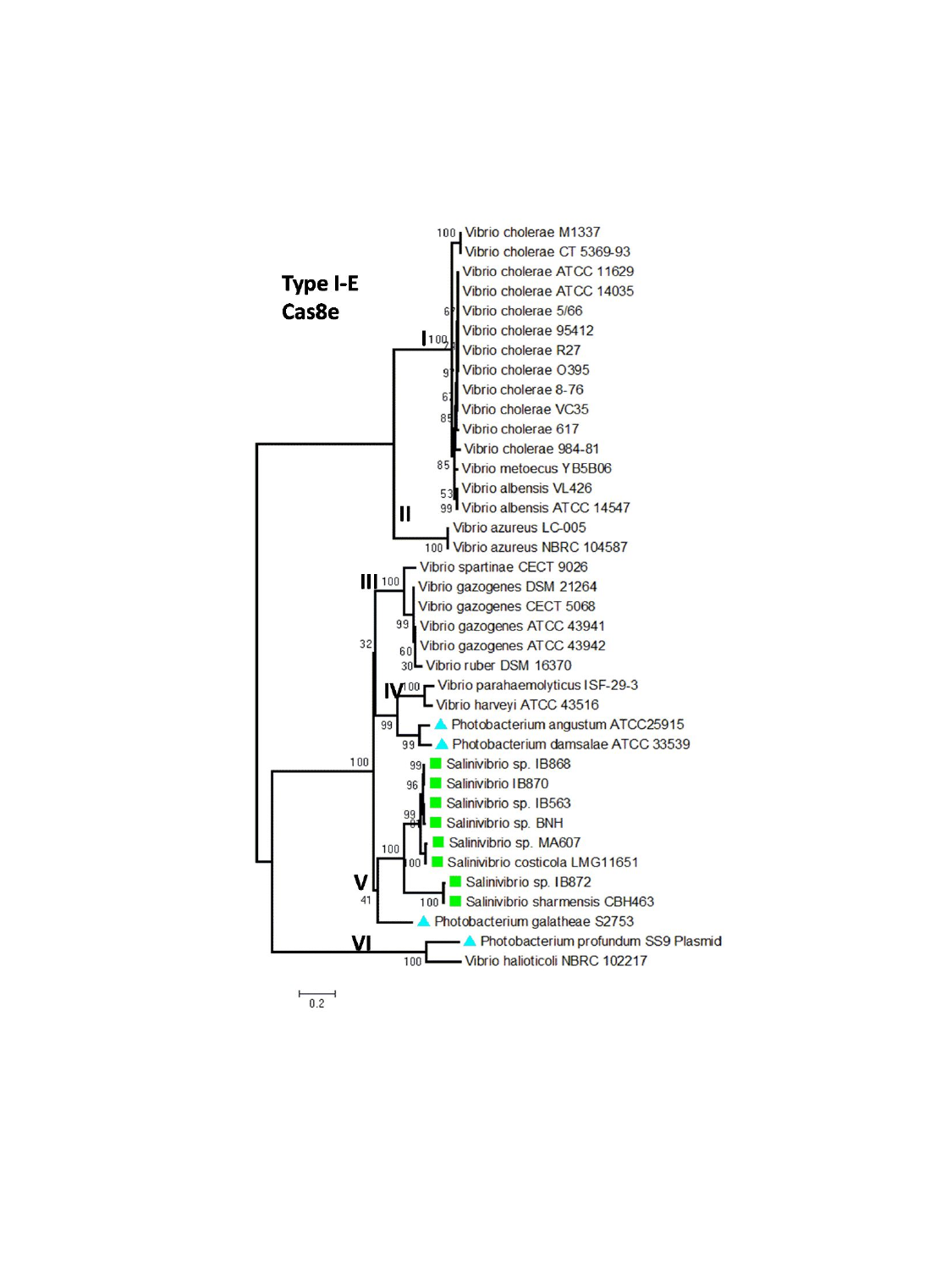

## Slide 8
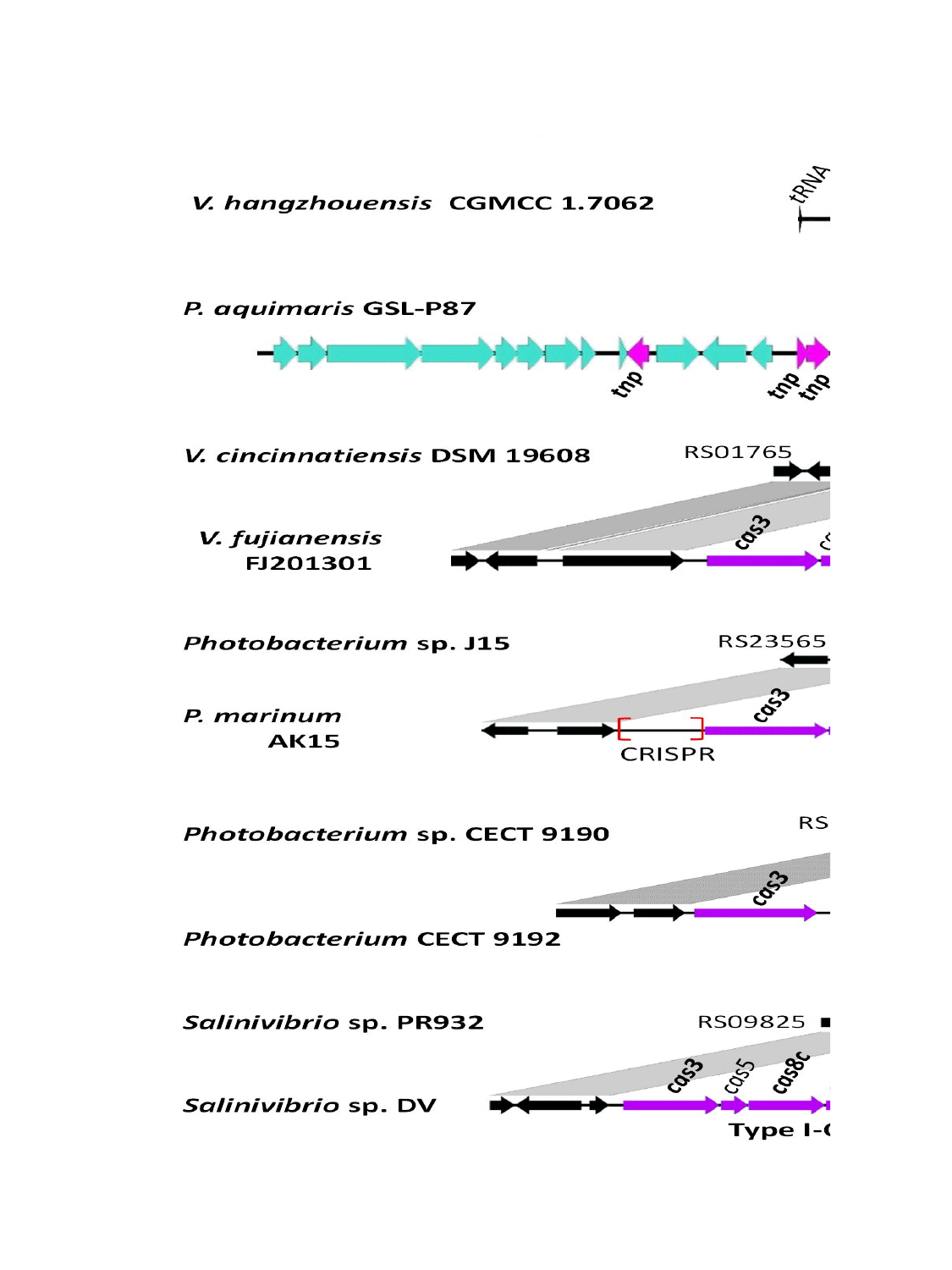

## Slide 9
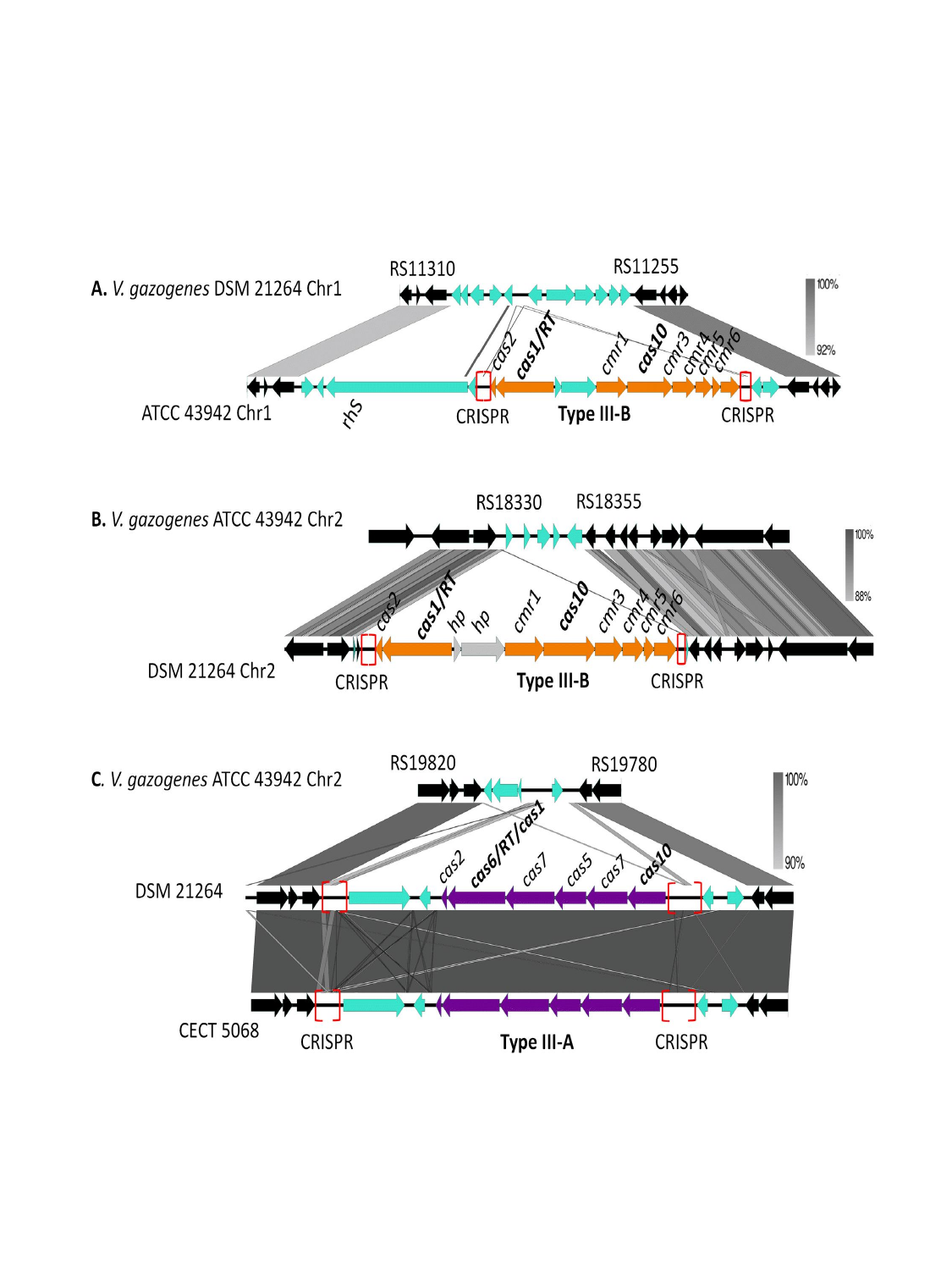

## Slide 10
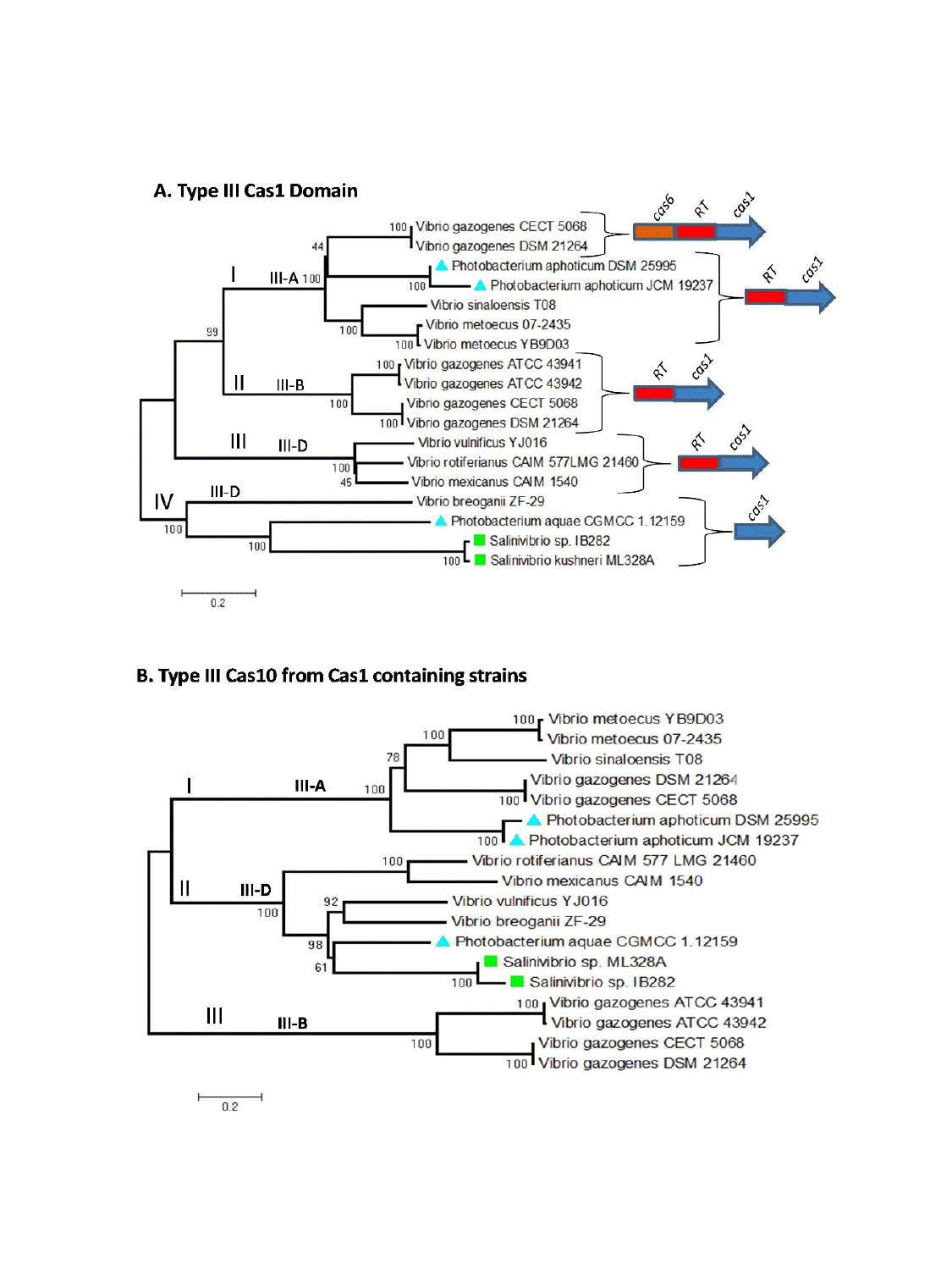

## Slide 11
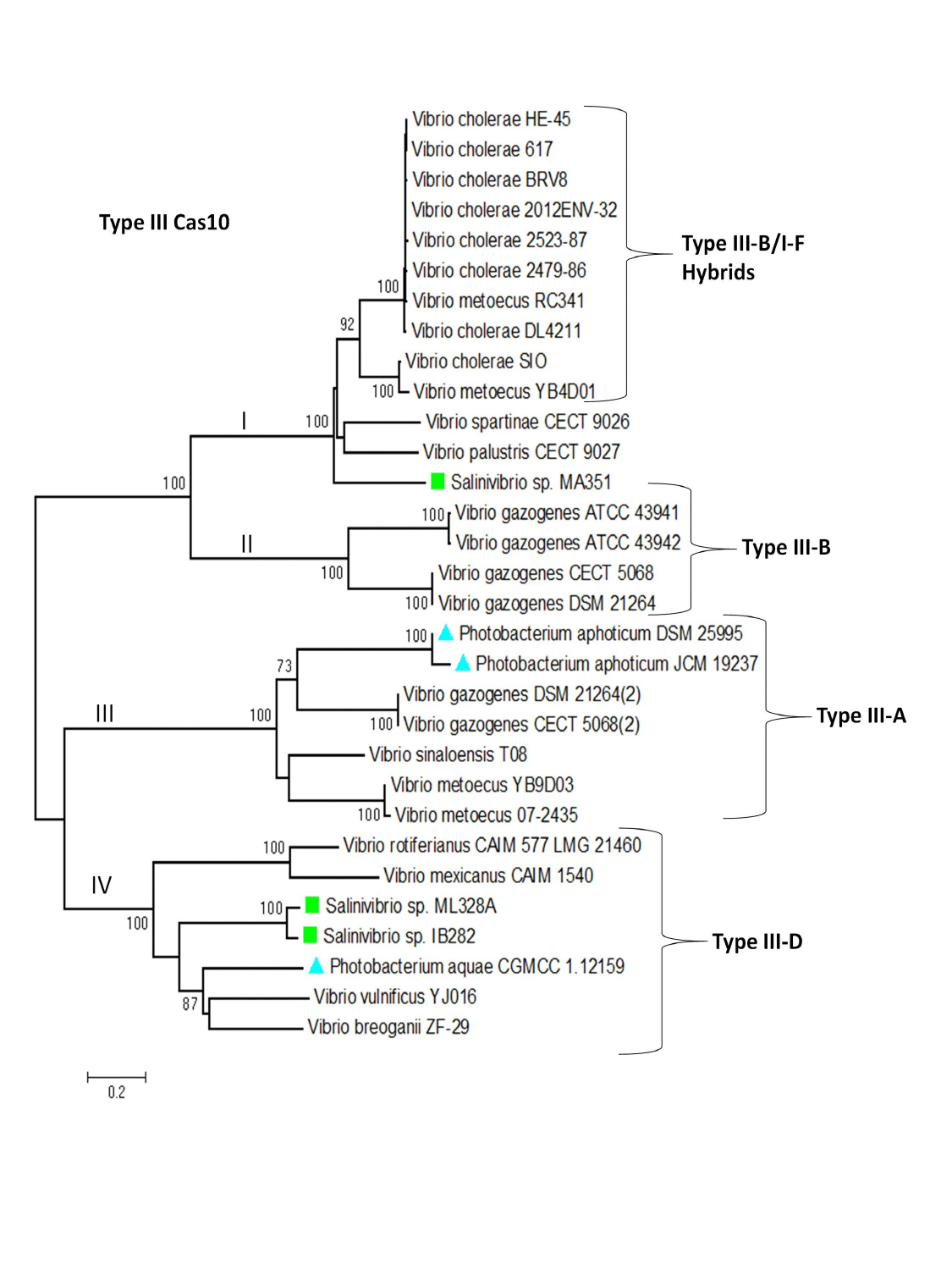

Supplement: Supplementary file 1 — Figure S1. Distribution of CRISPR-Cas system types identified among Vibrionaceae. (A) Percentage break down of all the systems identified by type. Different colors are used to indicate different CRISPR-Cas systems. (B) Percentage of systems identified that are associated with mobile genetic elements. (C) Diversity of each system identified within the Vibrionaceae. Figure S2. Phylogenetic analysis of cas1 and intV from V. cholerae strains containing type I-F system. The cas1 nucleotide sequences were examined for 21 type I-F systems characterized in V. cholerae. For the same 21 strains intV nucleotide sequences from VPI-6 were collected and analyzed. Aligned sequences were used to construct a neighbor-joining phylogenetic tree with bootstrap of 1000. Figure S3. Phylogenetic analysis of cmr1, cas6f and phage associated integrase. The nucleotide sequences for cmr1 and cas6f from V. cholerae and V. metoecus strains containing the putative type III-B/I-F system were examined to construct a phylogenetic tree. Similarly, the nucleotide sequences of the integrase from the prophage associated with the putative type III-B/I-F system were used to construct a phylogenetic tree. Aligned sequences were used to construct a neighbor-joining phylogenetic tree with bootstrap of 1000. Figure S4. Protospacer targets from V. cholerae I-F systems to Vibrio phages. Protospacer targets identified within the I-F arrays of Vibrio cholerae were mapped to the genomes of Vibrio phages X29/phi2, phage Kappa, phage fs2, fs-1, and KSF1-phi. Genomes were constructed using EasyFig. Figure S5. CRISPR-Cas type I-F systems in Vibrionaceae are within MGEs. Comparative analysis of the genomic regions containing type I-F systems demonstrated their presence on genomic islands. (A) The CRISPR-Cas island in V. metoecus YB5B04 is absent from V. metoecus strain OYP8G12. (B) The type I-F system present within an island in V. parahaemolyticus A4EZ703 between VPA0712 and VPA0713 relative to RIMD2210633 that lacks [file 12864_2019_5439_MOESM1_ESM.pptx]
